# Effects of fermentable fiber and polyphenol supplementation on mood and cognition in adults during hypobaric hypoxia exposure

**DOI:** 10.14814/phy2.70541

**Published:** 2025-09-10

**Authors:** Meaghan E. Beckner, Heather S. Fagnant, Philip J. Niro, Grace E. Giles, Harris R. Lieberman, J. Philip Karl

**Affiliations:** ^1^ Military Nutrition Division U.S. Army Research Institute of Environmental Medicine Natick Massachusetts USA; ^2^ Oak Ridge Institute for Science and Education Oak Ridge Tennessee USA; ^3^ U.S. Army Combat Capabilities Development Command Soldier Center Natick Massachusetts USA

**Keywords:** altitude, cognitive performance, dietary supplement, gut–brain axis, prebiotic

## Abstract

This study investigated the effects of fermentable fiber and polyphenol supplementation on mood and cognition following rapid ascent to simulated 4300 m. Healthy adults (*n* = 13, 21 ± 3 years) participated in a randomized, placebo‐controlled crossover study consisting of three, 2‐week phases separated by ≥1 week. Food products containing the fiber and polyphenol supplement or placebo were consumed during each phase. During the final 2 days of each phase, participants resided in a hypobaric chamber for 36 h simulating low (500 m) or high (4300 m) altitude, creating three conditions: placebo‐500 m, placebo‐4300 m, and supplement‐4300 m. A computerized cognitive test battery was administered each morning, evening, and ~36 h post‐chamber residence to assess mood and cognition. Total mood disturbance, tension, fatigue, and depression were higher, and total risk‐propensity score, energy, self‐control, and invincibility were lower at 4300 m versus 500 m independent of supplementation. Relative to placebo‐500 m, anger was higher during placebo‐4300 m, but not supplement‐4300 m, whereas confusion was greater during supplement‐4300 m but not placebo‐4300 m. More vigilance false alarms occurred during 500 m versus 4300 m independent of supplementation. Although short supplementation and washout periods may have limited observable effects, findings do not support favorable effects of this fiber and polyphenol supplement on cognition but suggest the supplement may attenuate hypobaric hypoxia‐induced increases in anger.

## 1 INTRODUCTION

The brain is a highly oxygen‐dependent organ, requiring over 20% of the body's total oxygen demand to maintain normal functioning (Aboouf et al., 2023). At high altitude (>2500 m above sea level), hypobaric hypoxia reduces neurotransmitter production and increases sympathoadrenal activity, reactive oxygen species (ROS), neuroinflammation, and blood brain barrier permeability (Aboouf et al., 2023; Bloomfield et al., 2023; McMorris et al., 2017; Pena et al., 2022; Song et al., 2016; Virues‐Ortega et al., 2004). Collectively, these physiological responses contribute to impaired mood and cognitive function following rapid ascent to high altitude, including increased depression and anxiety, slowed reaction time, and impaired memory and decision‐making (Heinrich et al., 2019; McMorris et al., 2017; Pighin et al., 2014; Shukitt & Banderet, 1988; Shukitt‐Hale et al., 1990, 1998; Shukitt‐Hale & Lieberman, 1996). These decrements can appear within 1 h and are most severe during the first 48 h at high altitude (Shukitt‐Hale et al., 1998). Acclimatization can improve mood and cognition (Shukitt‐Hale et al., 1998), but for populations such as military personnel or mountaineers, time to acclimate is not always possible before engaging in critical cognition‐dependent tasks. Although pharmacologic interventions to assist with adaptation at altitude are available, side effects limit their use (Department of the army, 2010). Identifying non‐pharmacologic interventions to mitigate the negative effects of high altitude on mood and cognitive performance could provide an alternative approach for optimizing performance in high altitude environments.

One such strategy may involve targeting the gut microbiota using nutrition‐based approaches. The gut microbiota produces neurotransmitters and secretes amino acids and other compounds such as short‐chain fatty acids (SCFA) that communicate with the central nervous system to influence mood, cognition, and stress regulation (Cryan & Dinan, 2012; Tooley, 2020; Yong et al., 2019). Prebiotics are substrates consumed in food or as dietary supplements that are selectively used by the gut microbiota conferring a health benefit (Gibson et al., 2017). Fermentable fibers such as inulin, galacto‐oligosaccharides [GOS], and resistant starch are among the most recognized prebiotics, having been shown to promote the growth of beneficial commensal gut bacteria such as *Bifidobacterium* spp. These prebiotics also provide substrate for the production of metabolites, and SCFAs in particular (Aslam et al., 2023; Freijy et al., 2022), which have immunomodulatory and anti‐inflammatory properties and may influence mood and cognition (Dalile et al., 2019).

Polyphenols are a class of dietary compounds found in fruits, vegetables, seeds, and nuts that also have “prebiotic‐like” effects (Berding et al., 2021; Hano & Tungmunnithum, 2020). Like fermentable fibers, polyphenols are metabolized and biotransformed by the gut microbiota into metabolites that can exert both anti‐inflammatory and antioxidant effects and may impact mood and cognition (Duda‐Chodak et al., 2015; Hano & Tungmunnithum, 2020; Kent et al., 2017; Pannu et al., 2021). Polyphenols can also impact mood and cognition independent of the gut microbiota by promoting vasodilation via nitric oxide production (Bell et al., 2015; Fisher et al., 2003) and enhancing parieto‐frontal brain connections (Schmidt et al., 2015). Indeed, acute cognitive benefits, including improved memory and attention, have been observed following consumption of cocoa flavanols, green tea extract, and berry anthocyanins, all rich polyphenol sources (Bell et al., 2015).

The effects of fermentable fiber supplementation on mood and cognition at high altitude are undetermined, and supplementation of individual polyphenols to improve cognitive function at high altitude (>4500 m) has only been investigated in animal models (Chen et al., 2023). Notably, the efficacy of both fiber and polyphenol interventions may vary by compound type, source, and dependency on gut microbial metabolism (Duda‐Chodak et al., 2015; Flint, 2012; Rastall, 2010). Therefore, co‐ingestion of diverse fermentable fiber and polyphenol sources may produce additive beneficial effects. In support, we previously demonstrated using an in vitro fermentation model that combining blends of three fermentable fibers (oligofructose‐enriched inulin, GOS, and high‐amylose corn starch) and four polyphenol sources (cocoa, green tea, blueberry, and cranberry) increased beneficial microbes, SCFA production, and antioxidant potential to a greater extent than either component alone (Whitman et al., 2024). In a subsequent study of healthy adults exposed to hypobaric hypoxia simulating 4300 m altitude, this fermentable fiber and polyphenol (FP) blend promoted beneficial gut microbes including *Bifidobacterium* spp., prevented hypobaric hypoxia‐induced increases in intestinal permeability, and attenuated decreases in peripheral oxygen saturation (SpO_2_), effects that could ameliorate high altitude‐induced mood and cognitive impairments (Karl et al., 2025). Evidence that the FP blend also increased SCFA production and reduced inflammation was supported by some, but not all, measurements (Karl et al., 2025). Herein, we report on a secondary objective of that study, which was to assess the effect of the FP supplement on mood and cognitive performance following rapid ascent to simulated 4300 m. We hypothesized that the FP supplement would attenuate hypobaric hypoxia‐induced decrements in mood and cognitive performance.

## 2 METHODS

### 2.1 Volunteers

Healthy men and women (age 17–39 years) were recruited to participate in this study from June 2019 through November 2022. Eligible participants had a body mass index (BMI) ≤ 30.0 kg/m^2^ and were recreationally active (aerobic and/or resistance exercise ≥3 days/week). Participants were excluded if born at altitudes >2100 m, living in areas >1200 m, or resided in areas >1200 m for ≥5 days within the previous 2 months. Additional exclusion criteria included a history of prior high altitude cerebral or pulmonary edema, a history of gastrointestinal disease or gastrointestinal surgery, pregnancy, musculoskeletal injuries, metabolic or cardiovascular disease, colonoscopy or use of antibiotics within 3 months of participation, anemia or Sickle Cell Trait, swallowing or sleeping disorders, regular use of medications, or following a vegetarian or vegan diet. Participants were instructed to discontinue any use of prebiotic, probiotic, or other dietary supplements at least 2 weeks prior to beginning study participation, to not consume probiotic‐containing foods during study participation, and to avoid alcohol, nicotine‐containing products, and caffeine during controlled diet periods.

The study was approved by the Headquarters U.S. Army Medical Research and Development Command Institutional Review Board. Investigators adhered to the policies regarding the protection of human subjects as prescribed in Army Regulation 70‐25, and the research was conducted in adherence with the provisions of 32 CFR Part 219 and the ethical principles of the Declaration of Helsinki. All participants provided written informed consent prior to participation and the trial was registered on www.clinicaltrials.gov as NCT04111263.

### 2.2 Experimental design

**FIGURE 1:**
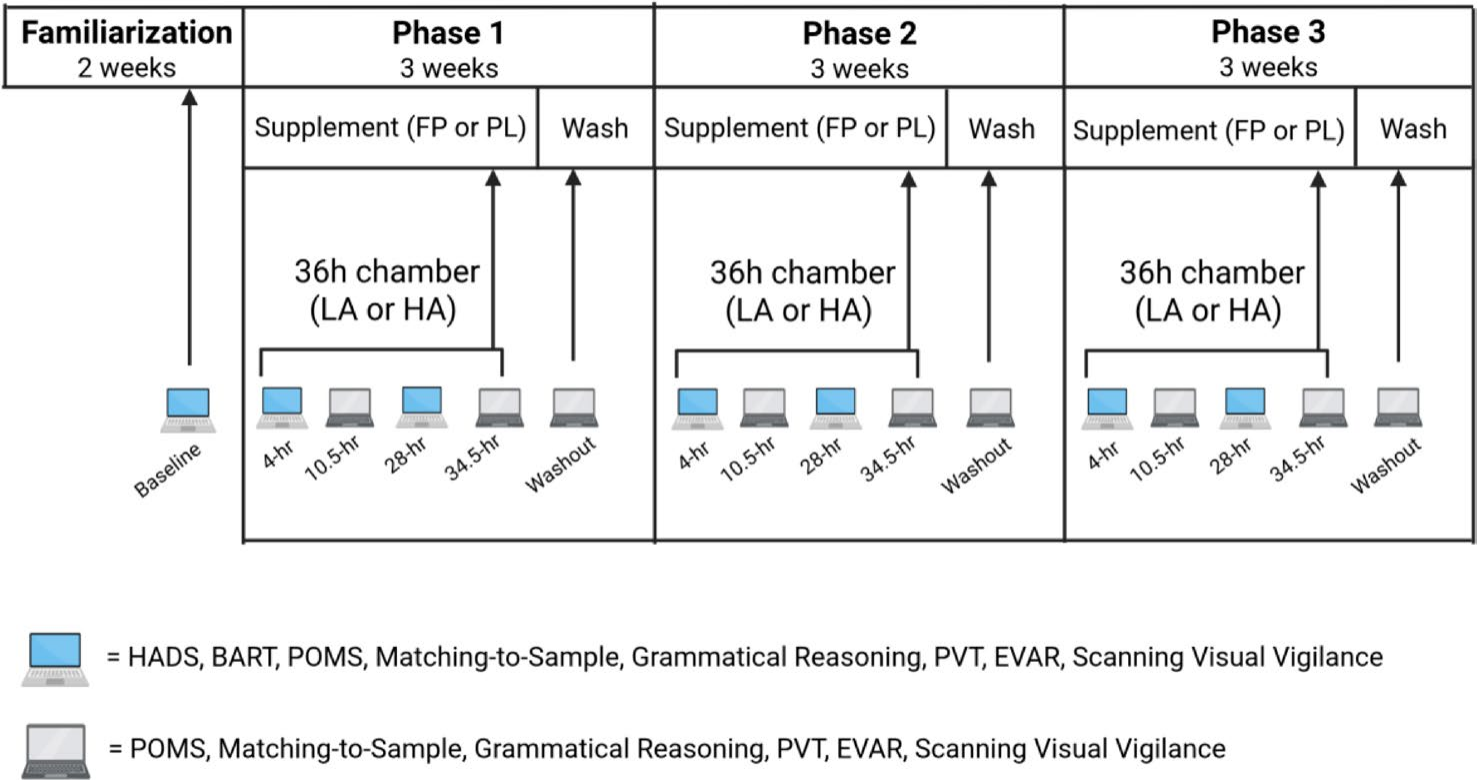
Experimental design. Participants completed familiarization testing and baseline mood and cognitive assessments (Baseline, 0700–0900 h) followed by three, 3‐week phases. Each phase included a 14‐day supplementation period followed by a ≥1‐week washout period (Wash). During each phase, participants consumed either a fermentable fiber and polyphenol (FP, one phase) or matched placebo (PL, two phases) supplement. During the final 2 days of each supplementation period, participants resided in a hypobaric chamber for 36 h simulating either low altitude (LA; 500 m, one phase) or high altitude (HA; 4300 m, two phases). Participants completed the mood and cognitive assessments after 4 h, 10.5 h, 28 h, and 34.5 h of chamber residence, and again ~36 h following the chamber residence (Washout). The combination of supplementation and altitude resulted in three experimental conditions: PL+LA, PL+HA, and FP+HA. BART, balloon analog risk task; EVAR, evaluation of risks scale; HADS, hospital anxiety and depression scale; POMS, profile of mood states; PVT, psychomotor vigilance test. Created in BioRender https://BioRender.com/kcx8hmc.

This randomized, placebo‐controlled crossover clinical trial consisted of three phases. Each phase included a 14‐day supplementation period followed by a ≥ 1‐week washout period (Figure 1). Participants consumed one of two types of supplemental snack bars during each supplementation period: fermentable fiber and polyphenol (FP, consumed during one phase) or a matched placebo (PL, consumed during two phases). The FP snack bar contained a blend of fermentable fibers and polyphenol sources (per 50 g snack bar): 1.38 g Orafti® Synergy1 (93.2% oligofructose‐enriched inulin by dry wt.; Beneo GmbH, Mannheim, Germany), 1.62 g Bimuno‐galactooligosaccharides (85% B‐GOS w/w; Clasado Biosciences, Reading, UK), 8.95 g Hi‐Maize 260 (59% resistant starch w/w; Ingredion, Inc., Bridgewater, New Jersey, USA), 300 mg cocoa seed extract (CocoActiv; 45.1% ± 2.6% total phenolics dry wt. catechin equivalents; Naturex; Avignon, France), 3.15 g wild blueberry powder (4.0% ± 0.1% total phenolics dry wt. gallic acid equivalents; Naturex), 220 mg cranberry extract (Cystricran®; 57.2% ± 1.3% total phenolics dry wt. gallic acid equivalents; Naturex), 125 mg green tea leaf extract (100% total phenolics dry wt. gallic acid equivalents; Naturex). The dosage of fermentable fibers was selected based on previous reports of doses that altered gut microbiota composition (So et al., 2018) with minimal gastrointestinal side effects (Grabitske & Slavin, 2009). Polyphenol sources and doses were selected to be representative of diverse groups of polyphenols in the diet and consistent with the upper levels of habitual dietary intake (Zamora‐Ros et al., 2016). Placebo products substituted maltodextrin for the fiber and polyphenol sources. The placebo product used during the low‐altitude condition differed slightly in color from the products used in the high‐altitude conditions so that investigators remained blinded to supplementation during the high‐altitude conditions. However, taste, energy, and macronutrient content were similar.

During Days 1–7 of each phase, participants consumed two snack bars daily during breakfast, equivalent to half the full dose of supplementation, to minimize side effects of increasing fiber intake. Snack bar consumption was monitored by study staff during all weekdays, and participants returned empty wrappers for bars consumed over weekends. During Days 8–14 of each phase, participants consumed four snack bars daily, two during breakfast, and two during lunch, and were provided a prescribed diet designed by registered dietitians and intended to maintain body weight. Consumption of breakfast, lunch, and the intervention snack bars was monitored by study staff. Participants were instructed to consume all snack bars, the entire prescribed diet, and nothing else except water, which was permitted ad libitum.

During Days 13–14 of each phase, participants resided in a hypobaric chamber for 36 h simulating either low altitude (LA; 500 m [720 mm Hg], one phase) or high altitude (HA; 4300 m [460 mm Hg], two phases). The hypobaric facility consisted of two chambers, one for sleeping (9 × 12 ft) and one for conducting testing procedures (9.7 × 20.6 ft), connected to an airlock to allow movement between chambers at the same pressure. Temperate (18–22°C, 20%–50% relative humidity) ambient conditions were maintained during all phases in the chamber. Participants were permitted to watch movies, read, or use their personal mobile phones between study‐related activities and were only permitted to sleep from 2000 to 0430 during the 36‐h chamber residence. The combination of supplementation and altitude resulted in three experimental conditions: PL+LA, PL+HA, and FP+HA. Conditions were group randomized so that cohorts of 2 to 4 participants were randomized to one of three experimental condition orders: (1) PL+LA, PL+HA, and FP+HA; (2) PL+HA, FP+HA, and PL+LA; (3) FP+HA, PL+LA, and PL+HA. Participants were blinded to chamber conditions and supplementation, while study investigators were blinded to supplementation during HA conditions only.

A self‐report mood questionnaire and battery of cognitive performance tests were administered during each chamber residence to measure secondary study outcomes (Figure 1). The cognitive test battery assessed a wide range of cognitive functions from simple (e.g., vigilance) to complex functions (e.g., logical reasoning) that are sensitive to the effects of nutritional interventions, high altitude, and military training (Beckner et al., 2023; Lieberman et al., 2016; Shukitt‐Hale et al., 1998). During the 2 weeks preceding the first phase, participants completed three familiarization sessions of the cognitive test battery between 0700 and 0900 h and the third familiarization session served as baseline. The Hospital Anxiety and Depression Scale (HADS) and balloon analog risk task (BART), described below, were completed once during the week prior to the first phase for familiarization. The mood and cognitive performance assessments were administered after 4, 10.5, 28 and 34.5 h of chamber residence. The HADS and BART were completed after 4 and 28 h of chamber residence only. All mood and cognitive assessments, except HADS and BART, were completed again ~36‐h following the chamber residence (washout).

### 2.3 Mood and cognitive assessments

The mood and cognitive assessments were administered on Dell Latitude Windows E5520 laptop computers using a custom software package provided by G‐Squared Software, Inc. (Toronto, ON, Canada) or a custom‐built executable by the research team (BART). The order of tests was identical during each session: HADS, BART, Profile of Mood States (POMS), Matching‐to‐Sample, Grammatical Reasoning, Psychomotor Vigilance Test (PVT), Evaluation of Risks Questionnaire (EVAR), and Scanning Visual Vigilance Task.

### 2.4 Hospital anxiety and depression scale (HADS)

The HADS is a scale used to quantify depression and anxiety symptoms within the past week (Zigmond & Snaith, 1983). The HADS contains 14 self‐report statements in which participants rate attitudes and thoughts associated with depression and anxiety on a scale from 0 (no impairment) to 3 (severe impairment). Outcomes include the scores for depression and anxiety, each ranging from 0 to 21.

### 2.5 Balloon analog risk task (BART)

The BART assesses risk‐taking behavior (Lejuez et al., 2002). Participants were presented with a simulated balloon and instructed to inflate the balloon to a desired level. With each pump, a mock $0.01 was accrued in a temporary reserve hidden from the participant. At any point during each balloon trial, the participant may stop inflating the balloon and transfer the money to a permanent bank. If the balloon explodes, all the money in the temporary reserve is lost. Each balloon has a 1/8, 1/32, or 1/128 probability of exploding, which is unknown to the participant. The task includes 90 trials (30 for each probability) with the objective to collect as much money as possible. Outcomes included the adjusted average number of pumps for all unexploded balloons and for each balloon probability, with higher scores indicative of greater risk‐taking behavior.

### 2.6 Profile of mood states (POMS)

The Profile of Mood States 2nd edition (POMS 2‐A) questionnaire is a 65‐item inventory of adjectives divided into seven subscales (tension, depression, anger, vigor, fatigue, confusion, and friendliness) that participants used to self‐report mood states (Heuchert & McNair, 2012). The POMS is sensitive to a variety of nutritional manipulations and environmental factors, such as undernutrition, physical activity, and sleep loss (Karl et al., 2015; Lieberman et al., 2005). A total mood disturbance score was calculated by summing the scores from the tension, depression, anger, fatigue, and confusion subscales and subtracting the vigor subscale score, with higher values indicative of greater mood disturbance; friendliness is not included in the total mood disturbance score (Heuchert & McNair, 2012).

### 2.7 Matching to sample

The Matching to Sample Test assesses short‐term spatial memory (working memory) and pattern recognition skills (Mahoney et al., 2007). Participants were presented with an 8 X 8 matrix of a red and green checkerboard on a color screen for 4 s. The matrix was then removed and followed by a variable delay interval (either 8 or 16 s) during which the screen was blank. After the delay, two matrices were presented on the screen: the original sample matrix and a second matrix that differed slightly. The participant was asked to identify the original matrix within 15 s, otherwise a time‐out error was recorded. Outcomes included the number of lapses (i.e., no response within 15 s), the number of correct responses, and mean correct response time for all 24 trials.

### 2.8 Grammatical reasoning

The Grammatical Reasoning Test assesses language‐based logical reasoning and has been used to assess the effects of various treatments on cognitive function (Dorrian et al., 2003; Lieberman et al., 2016). The test was adapted from the Baddeley Grammatical Reasoning Test and consists of 32 trials (Baddeley, 1968). For each trial, the letters AB or BA followed a statement. The participant decided whether each statement correctly described the order of the two letters. Outcomes included the number of lapses (i.e., no response within 500 msec), the number of correct responses, and mean correct response time.

### 2.9 The psychomotor vigilance test (PVT)

The psychomotor vigilance test (PVT) measures simple visual reaction time and is particularly sensitive to the vigilance decrements associated with sleep disruption and other environmental factors (Dinges & Powell, 1985). The participant responded as quickly as possible to a series of stimuli presented at random intervals on a screen for a total duration of 10 min. The total number of stimuli presented varied per administration based on the participant's response time. Responses made before or after stimulus occurrence were recorded as false alarms. Outcomes included percentage of false alarms, percentage of lapses (i.e., no response within 500 msec), and percentage of hits, based on the total number of stimuli presented, as well as mean correct response time.

### 2.10 Evaluation of risks scale (EVAR)

The Evaluation of Risks (EVAR) Questionnaire measures willingness to take risks through participant responses to 24 items using a visual analog scale anchored by descriptors such as “not at all” and “very much” (Killgore et al., 2006; Sicard et al., 1999). The scale has been shown to differentiate individuals who routinely engage in risky behavior, and it also correlates with measures of sensation‐seeking and other risk‐related traits (Killgore et al., 2006). The scale yielded five factors including self‐control, danger‐seeking, energy, impulsiveness, invincibility, and a total risk‐taking propensity score.

### 2.11 Scanning visual vigilance

The Scanning Visual Vigilance Task measures simple visual vigilance and is designed to simulate various critical activities that require maintenance of vigilance, such as radar and sonar watch (Fine et al., 1994). The participant responded as quickly as possible to a faint stimulus that appeared randomly on a computer screen for 2 s, occurring approximately once a minute. A total of 30 stimuli were presented. Outcomes included the number of false alarms, lapses (i.e., no response within 500 msec), valid responses, and mean correct response time.

### 2.12 Statistical analysis

Power analysis was performed using GLIMMPSE v.2.2.7 (http://glimmpse.samplesizeshop.org/#/). Sample size calculations were based on previously reported impairments in working memory at HA (≥4500 m) compared to sea level (de Aquino Lemos et al., 2012; Malle et al., 2016). Using means and standard deviations from these studies, *n* = 15 was determined to be sufficient to detect a small to medium effect size for the FP supplementation at *α* = 0.05 and power = 0.80.

Cognitive performance and mood data were analyzed using linear mixed models. Experimental condition (PL+LA, PL+HA, and FP+HA), time, and their interaction were included as fixed factors. Study phase (first, second, and third) and experimental condition order were also included as fixed factors in the model. Participant was included as a random intercept. Baseline mood and cognitive performance, except BART, were included as covariates. Least significant difference post hoc testing was used when significant main effects or interactions were identified to determine between‐ and within‐group differences. Residuals were examined to verify assumptions of normal distribution and homogeneity of variance were met. All POMS subscales, except friendliness, were not normally distributed and were square root‐transformed for analysis. The primary analysis included the 13 participants who completed all three study phases (complete case (CC) cohort). A secondary analysis was conducted for the 26 participants who completed at least one study phase (intention‐to‐treat (ITT) cohort). Results from the CC analyses are presented in the main text and figures, with results from the ITT analyses provided in the supplemental material. Results that differed between CC and ITT are noted in the text. For all statistical tests, a *p* value of ≤0.05 was considered statistically significant. All analyses were conducted using SPSS (version 28; IBM SPSS Inc., Chicago, IL). Exploratory analyses conducted post hoc to support interpretation of the observed mood outcomes were conducted using repeated measures correlation analyses (Bakdash & Marusich, 2017) with the rmcorr (https://CRAN.R‐project.org/package=rmcorr) package in R (version 4.4.1; R Core Team, Vienna, Austria).

## 3 RESULTS

Thirty‐three participants volunteered and were determined eligible to participate. Two withdrew during familiarization for personal reasons, 2 withdrew due to GI symptoms during the first study phase, and three were withdrawn during the first study phase due to institution‐wide shutdowns related to the COVID‐19 pandemic (Figure S1). The remaining 26 participants completed at least one study phase (ITT cohort, Table 1). Of these participants, nine were withdrawn due to institution‐wide shutdowns related to the COVID‐19 pandemic, one withdrew due to GI symptoms at altitude during PL+HA, one withdrew due to relocation from the study area, one withdrew for personal reasons, and one did not complete chamber testing during FP+HA due to an unrelated illness. The remaining 13 participated in all three phases (CC cohort, Table 1), although three did not complete the second day of chamber residence during FP+HA due to acute mountain sickness (AMS)‐related symptoms. All 13 participants were included in analyses of self‐report measures (HADS, POMS, and EVAR), but one participant was excluded from the cognitive performance analyses due to nonadherence to test instructions. Median adherence to snack bar consumption was 100% across the three experimental conditions.

**TABLE 1:** Baseline participant characteristics.

	ITT cohort completed ≥1 chamber residence	CC cohort participated in 3 chamber residences
Male, Female (*n*)	24, 2	12, 1
Age (y)	22 ± 3	21 ± 3
Height (cm)	175.4 ± 7.2	175.5 ± 7.0
Body mass (kg)	78.3 ± 9.3	78.1 ± 8.7
BMI (kg/m^2^)	25.4 ± 2.5	25.4 ± 2.4

*Note*: Data presented as mean ± standard deviation.

*Abbreviations*: BMI, body mass index; CC, complete case; ITT, intention‐to‐treat.

Primary study outcomes are reported elsewhere (Karl et al., 2025). Briefly, the FP altered gut microbiota composition by decreasing α‐diversity, increasing relative abundance of *Bifidobacterium* spp. and *Ruminococcus* spp., and altering relative abundances of several other genera. FP supplementation did not alter fecal SCFA concentrations, but did reduce colonic pH, which is consistent with increased microbiota‐derived SCFA production. FP supplementation also prevented hypobaric hypoxia‐induced increases in intestinal permeability within the small intestine and proximal colon and circulating interleukin‐6 concentrations. Somewhat unexpectedly, FP supplementation attenuated hypoxia‐induced decreases in SpO_2_ with mean oxygen saturation during HA exposure being ~2% higher during FP relative to PL. However, FP supplementation also increased gastrointestinal symptoms and the incidence and severity of acute mountain sickness relative to PL.

Summary data for all cognitive outcomes are provided in Tables S1–S8.

### 3.1 HADS

There were no conditions, time, or interaction effects for depression or anxiety symptoms (Figure S2).

### 3.2 BART

**FIGURE 2:**
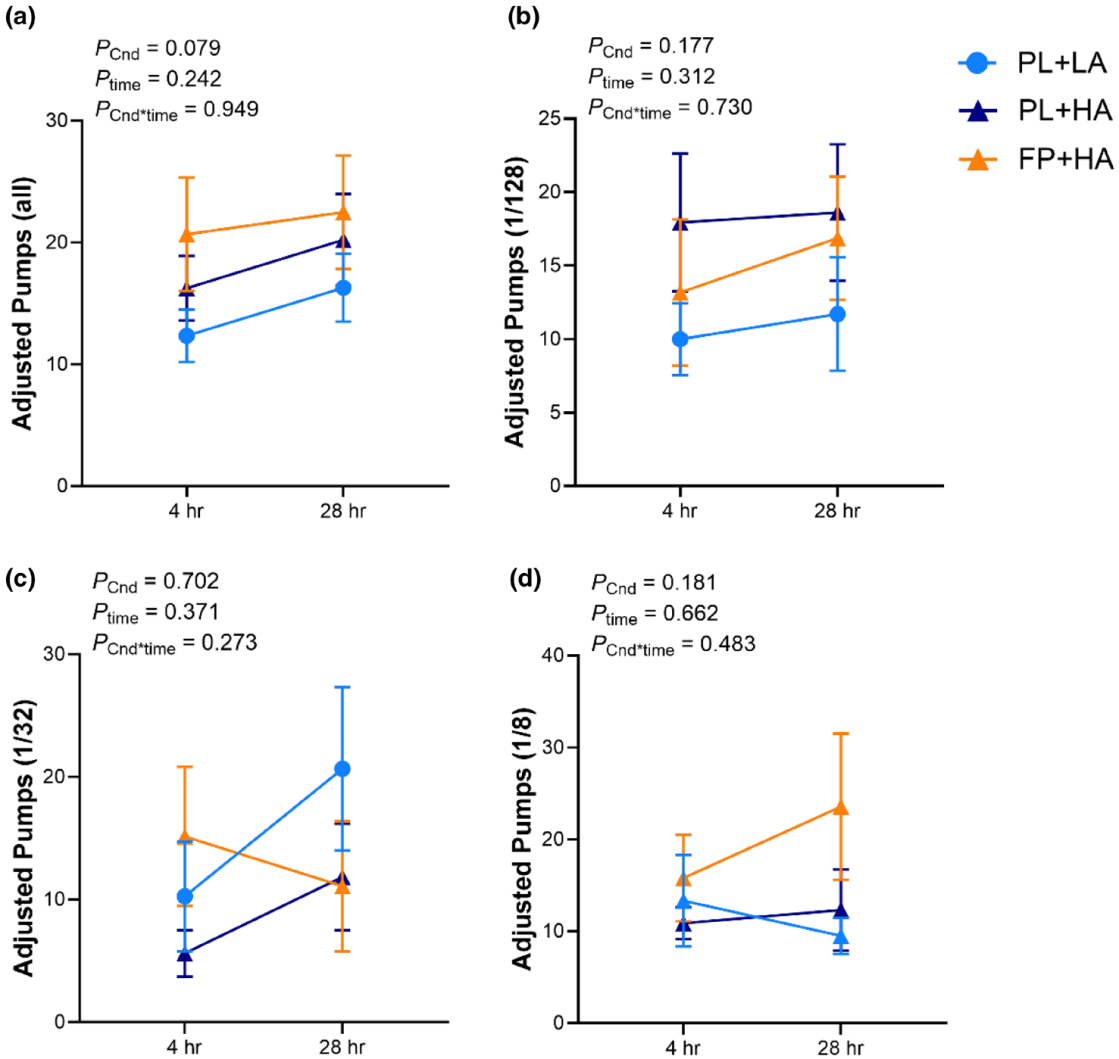
Balloon analog risk task (BART). BART was completed after 4 h and 28 h of hypobaric chamber residence at low altitude (LA; 500 m) or high altitude (HA; 4300 m) with daily consumption of a fiber and polyphenol (FP) or matched placebo (PL) snack bar (*n* = 12). Line graph represents raw data as the mean and standard error. Exact *p* values are presented within the figures for the main effect of experimental condition (*p*_Cnd_), main effect of time (*p*_time_), and their interaction (*p*_Cnd*time_).

The adjusted number of pumps for all balloon trials or by exploding probability did not differ across experimental conditions in the CC analysis (Figure 2a–d). In the ITT analysis, the adjusted number of pumps was greater during FP+HA versus PL+LA, but not PL+HA, for all balloon trials and the 1/128 probability balloons (Figure S3a,b). The adjusted number of pumps for 1/32 probability balloons was lower during FP + HA versus PL+LA, but not PL+HA, at 28 h of chamber residence (Figure S3c). There were no effects of HA or FP intervention on the adjusted number of pumps in the 1/8 probability (Figure S3d).

### 3.3 POMS

**FIGURE 3:**
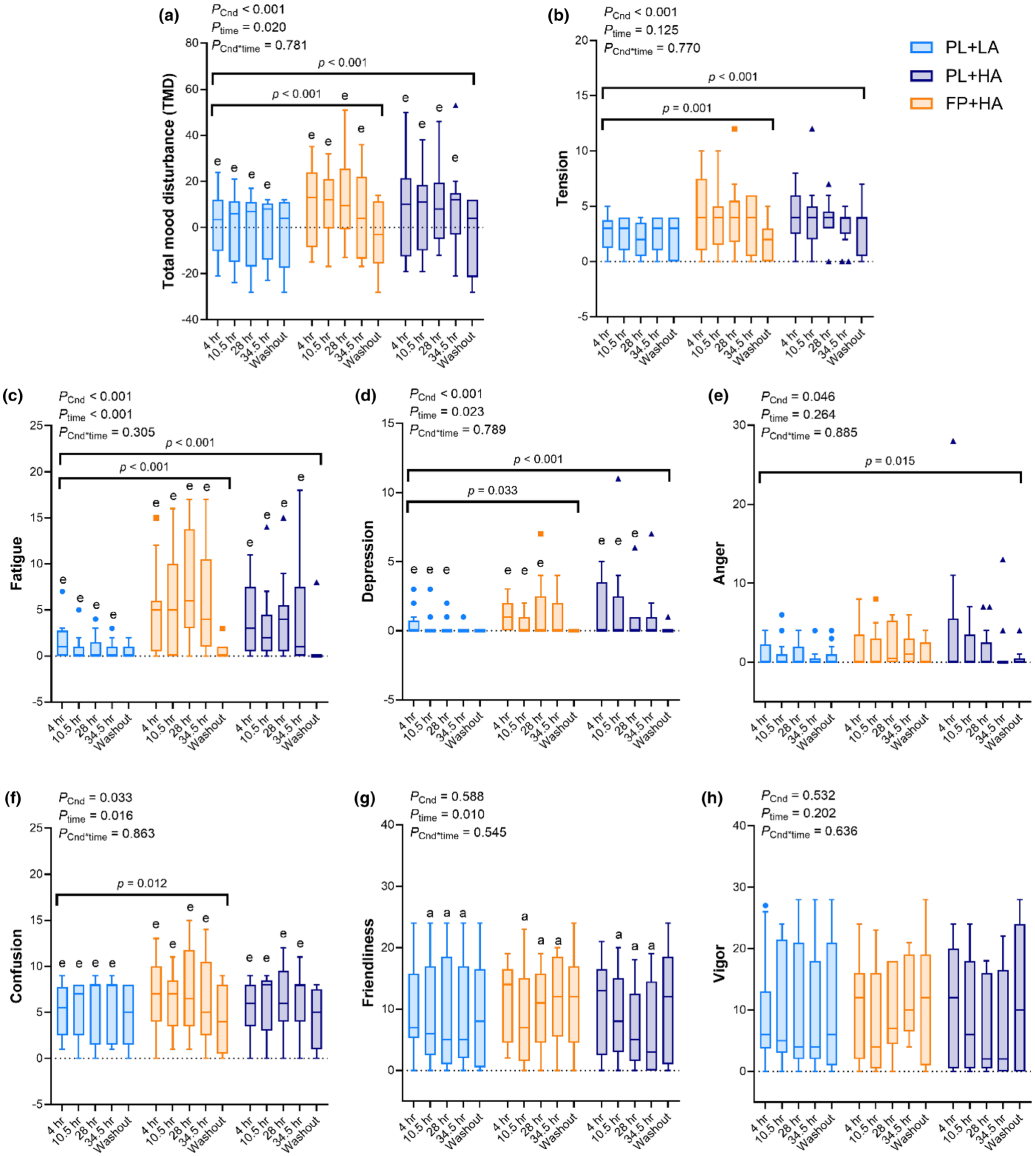
Profile of mood states (POMS). Profile of mood states was measured after 4 h, 10.5 h, 28 h, and 34.5 h of hypobaric chamber residence at low altitude (LA; 500 m) or high altitude (HA; 4300 m) and 36 h post‐chamber (Washout) with daily consumption of a fiber and polyphenol (FP) or matched placebo (PL) snack bar (*n* = 13). All POMS subscales except friendliness were square root transformed for analysis. Boxes indicate raw data median and interquartile range. Whiskers extend to 1.5 times the interquartile range, or to the minimum and maximum if no outliers are present. Outliers are presented as individual data points. No significant interaction effects were observed. Post hoc *p* values following significant main effect of experimental condition (*) are presented above horizontal bars. Letters represent least significant difference post hoc pairwise comparisons for main effect of time. a = significantly (*p* < 0.05) different than 4 h; e = significantly different than Washout. Exact *p* values are presented within the figures for main effect of experimental condition (*p*_Cnd_), main effect of time (*p*_time_), and their interaction (*p*_Cnd*time_).

Total mood disturbance, tension, fatigue, and depression were higher during both HA conditions versus LA (*p* < 0.05), with no difference between PL+HA and FP+HA (Figure 3a–d, Figure S4a–d). Anger was higher during PL+HA versus PL+LA (*p* = 0.015) but was not significantly different during FP+HA versus PL+LA (Figure 3e, Figure S4e). Confusion was greater during FP+HA versus PL+LA (*p* = 0.012), but not versus PL+HA (Figure 3f, Figure S4f). There were no effects of HA or FP intervention on friendliness or vigor (Figure 3g,h; Figure S4g,h). Exploratory repeated measures correlation analyses revealed a positive association between depression and anger across timepoints (*r*_rm_(157) = 0.478, *p* < 0.001).

### 3.4 Matching to sample

**FIGURE 4:**
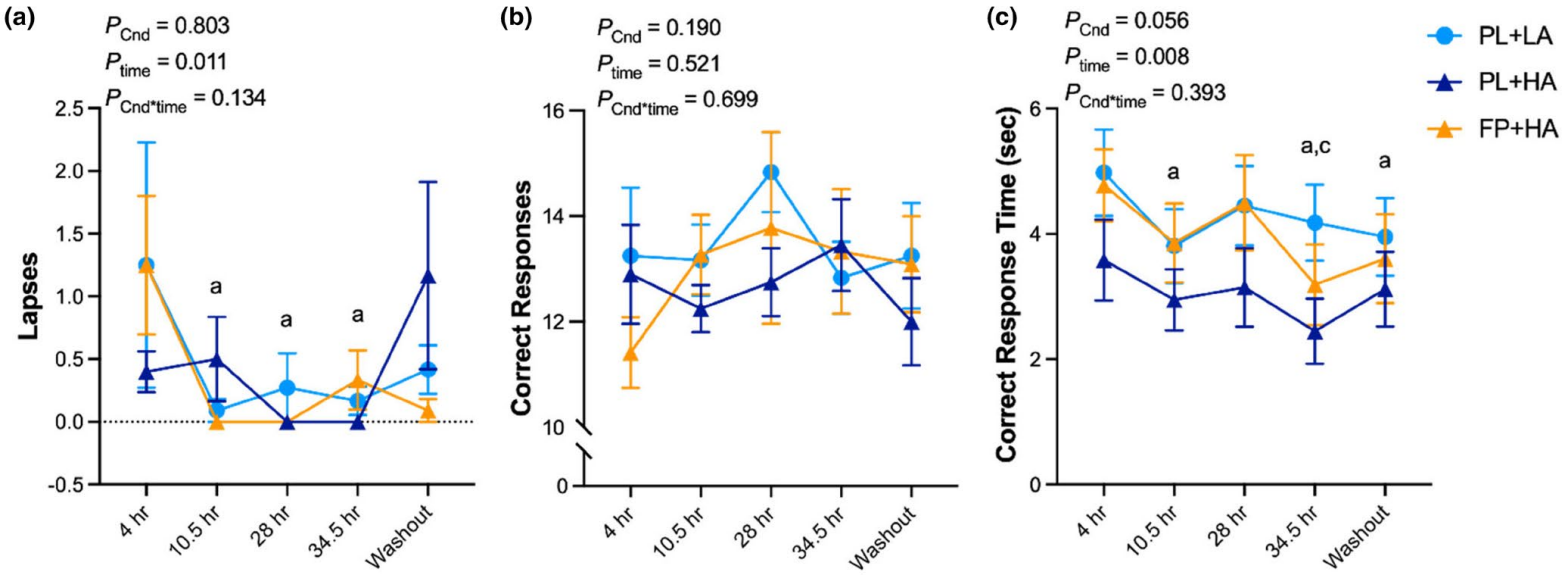
Matching to sample. The matching to sample test was administered after 4 h, 10.5 h, 28 h, and 34.5 h of hypobaric chamber residence at low altitude (LA; 500 m) or high altitude (HA; 4300 m) and 36 h post‐chamber (Washout) with daily consumption of a fiber and polyphenol (FP) or matched placebo (PL) snack bar (*n* = 12). Line graph represents raw data as mean and standard error. No significant condition or interaction effects were observed. Letters represent least significant difference post hoc pairwise comparisons for the main effect of time. a = significantly (*p* < 0.05) different than 4 h; c = significantly different than 28 h. Exact *p* values are presented within the figures for the main effect of experimental condition (*p*_Cnd_), main effect of time (*p*_time_), and their interaction (*p*_Cnd*time_).

The number of lapses and correct responses did not differ across experimental conditions (Figure 4a,b; Figure S5a,b). Mean correct response time demonstrated a tendency to differ across experimental conditions in the CC analysis (Figure 4c) and was faster during PL+HA versus PL+LA, but not FP+HA, in the ITT analysis (Figure S5c).

### 3.5 Grammatical reasoning

**FIGURE 5:**
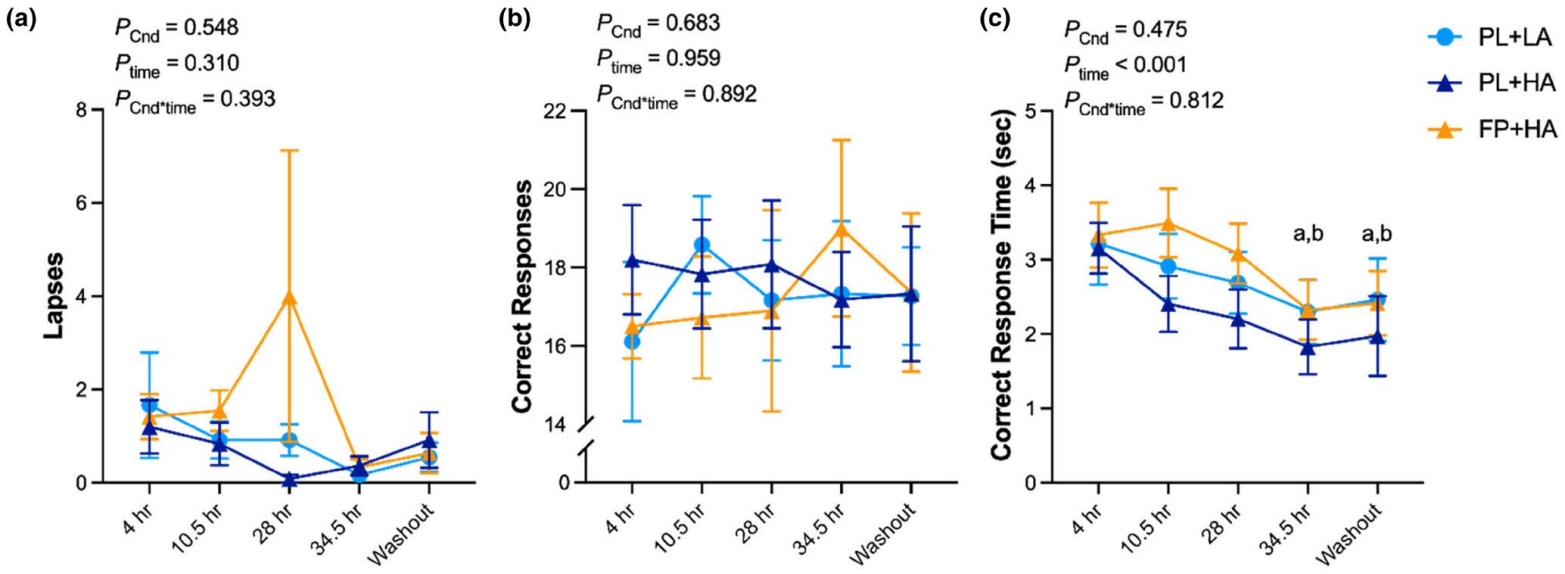
Grammatical reasoning. The grammatical reasoning test was administered after 4 h, 10.5 h, 28 h, and 34.5 h of hypobaric chamber residence at low altitude (LA; 500 m) or high altitude (HA; 4300 m) and 36 h post‐chamber (Washout) with daily consumption of a fiber and polyphenol (FP) or matched placebo (PL) snack bar (*n* = 12). Line graph represents raw data as mean and standard error. No significant condition or interaction effects were observed. Letters represent least significant difference post hoc pairwise comparisons for the main effect of time. a = significantly (*p* < 0.05) different than 4 h; b = significantly different than 10.5 h. Exact *p* values are presented within the figures for the main effect of experimental condition (*p*_Cnd_), main effect of time (*p*_time_), and their interaction (*p*_Cnd*time_).

There were no significant differences in the number of lapses, correct responses, or mean correct response times across experimental conditions (Figure 5a–c; Figure S6a–c).

### 3.6 PVT

**FIGURE 6:**
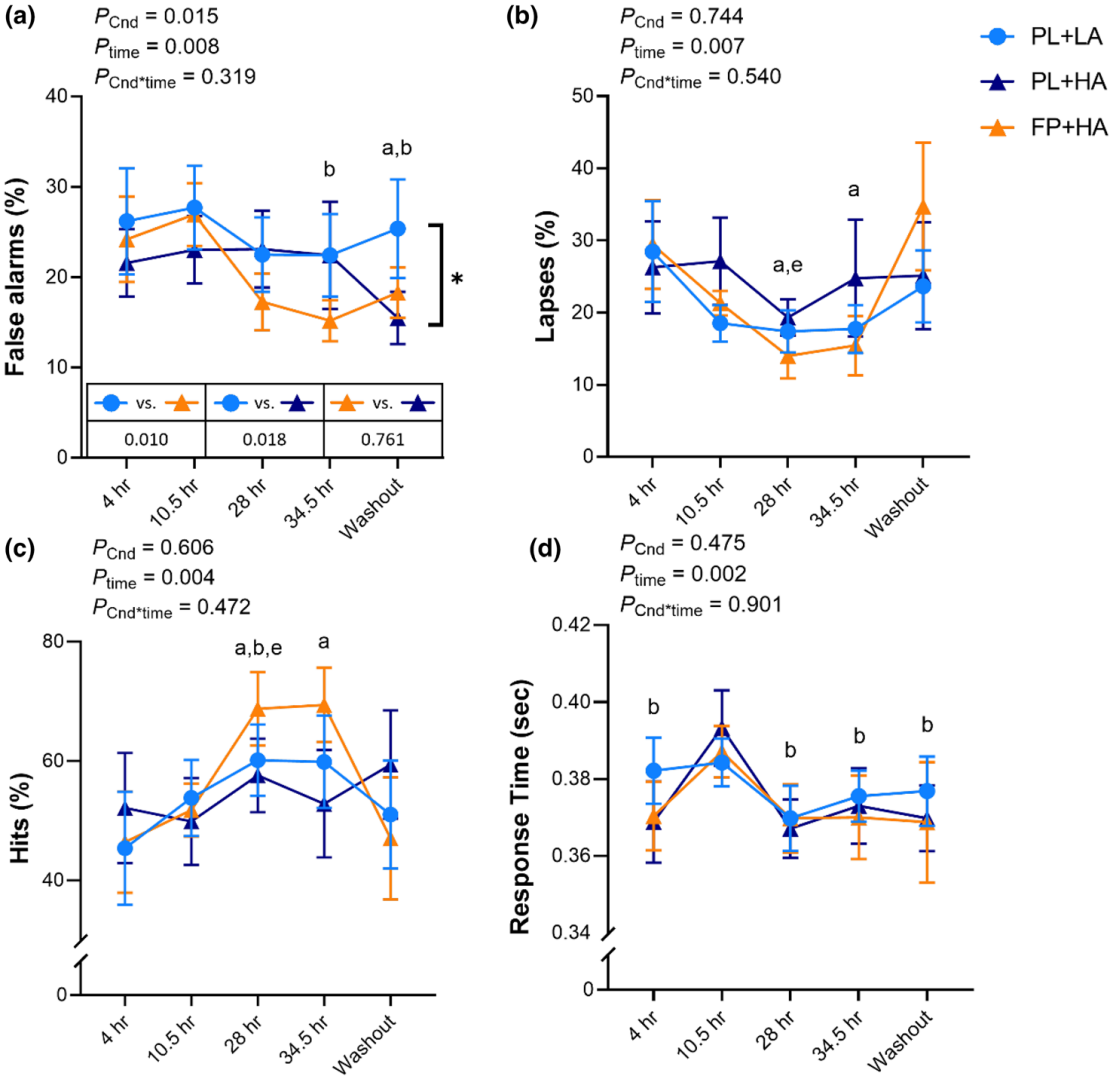
Psychomotor vigilance test (PVT). Psychomotor vigilance was assessed after 4 h, 10.5 h, 28 h, and 34.5 h of hypobaric chamber residence at low altitude (LA; 500 m) or high altitude (HA; 4300 m) and 36 h post‐chamber (Washout) with daily consumption of a fiber and polyphenol (FP) or matched placebo (PL) snack bar (*n* = 12). Line graph represents raw data as mean and standard error. No significant interaction effects were observed. *Main effect of experimental condition (*p* < 0.05) with post hoc *p* values presented within the embedded table. Letters represent least significant difference post hoc pairwise comparisons for the main effect of time. a = significantly (*p* < 0.05) different than 4 h; b = significantly different than 10.5 h; e = significantly different than Washout. Exact *p* values are presented within the figures for the main effect of experimental condition (*p*_Cnd_), main effect of time (*p*_time_), and their interaction (*p*_Cnd*time_).

Participants had a greater percentage of false alarms during PL+LA than both HA conditions, with no difference between PL+HA and FP+HA, in the CC analysis (Figure 6a). No differences in false alarms were observed in the ITT analysis (Figure S7a). The proportions of lapses and hits and response time did not differ by experimental condition (Figure 6b–d; Figure S7b–d).

### 3.7 EVAR

**FIGURE 7:**
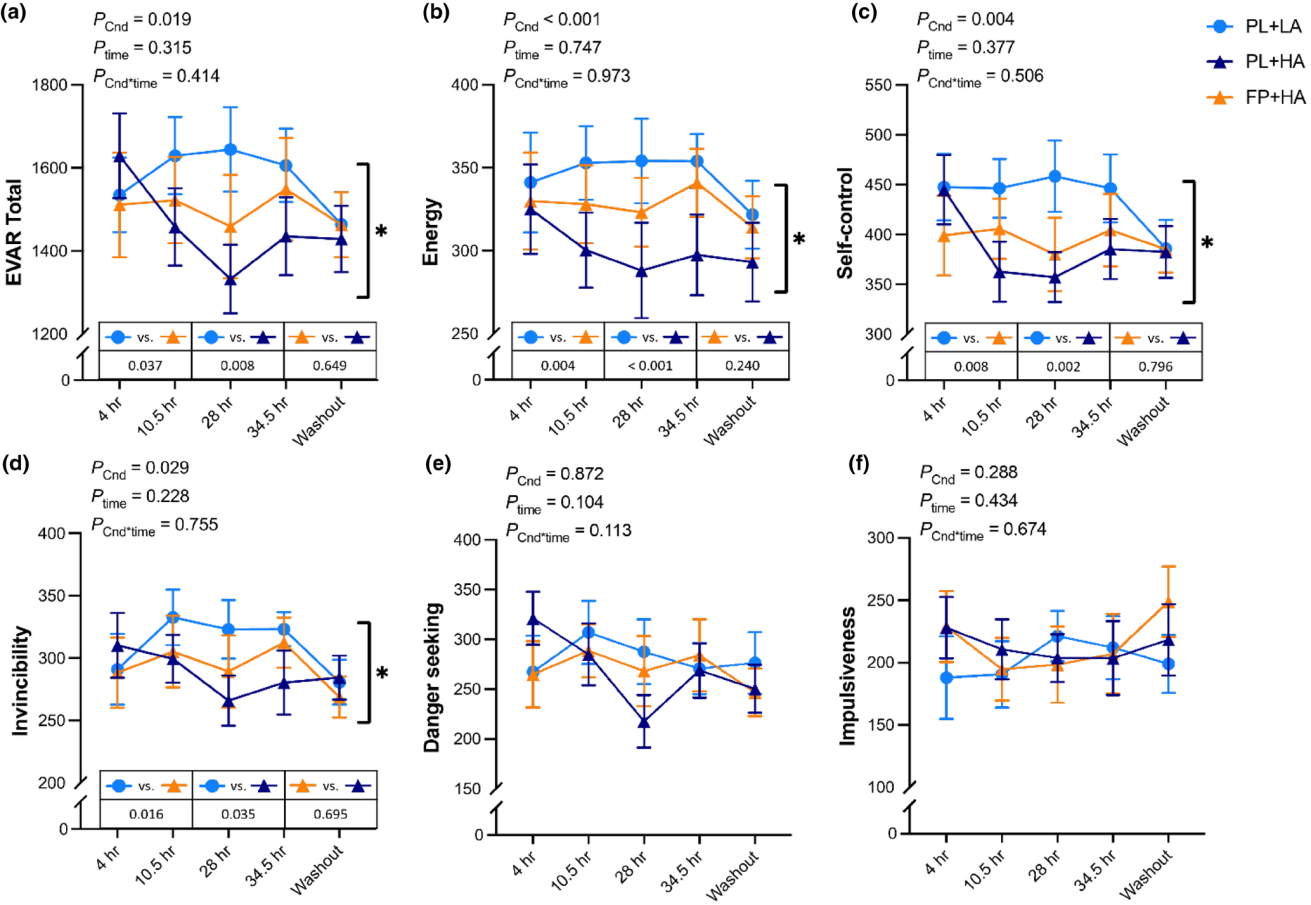
Evaluation of risks scale (EVAR). The EVAR scale was administered after 4 h, 10.5 h, 28 h, and 34.5 h of hypobaric chamber residence at low altitude (LA; 500 m) or high altitude (HA; 4300 m) and 36 h post‐chamber (Washout) with daily consumption of a fiber and polyphenol (FP) or matched placebo (PL) snack bar (*n* = 13). Line graph represents raw data as mean and standard error. No significant time or interaction effects were observed. *Main effect of experimental condition (*p* < 0.05) with post hoc *p* values presented within the embedded table. Exact *p* values are presented within the figures for main effect of experimental condition (*p*_Cnd_), main effect of time (*p*_time_), and their interaction (*p*_Cnd*time_).

Total risk‐propensity score, energy, self‐control, and invincibility were lower during both HA conditions versus LA (*p* < 0.05), with no differences between PL+HA and FP+HA (Figure 7a–d; Figure S8a–d). There were no significant differences in danger‐seeking or impulsiveness across experimental conditions (Figure 7e,f; Figure S8e,f). Exploratory repeated measures correlation analyses revealed a negative association between POMS anger and EVAR energy (*r*_rm_(157) = −0.204, *p* = 0.001).

### 3.8 Scanning visual vigilance

**FIGURE 8:**
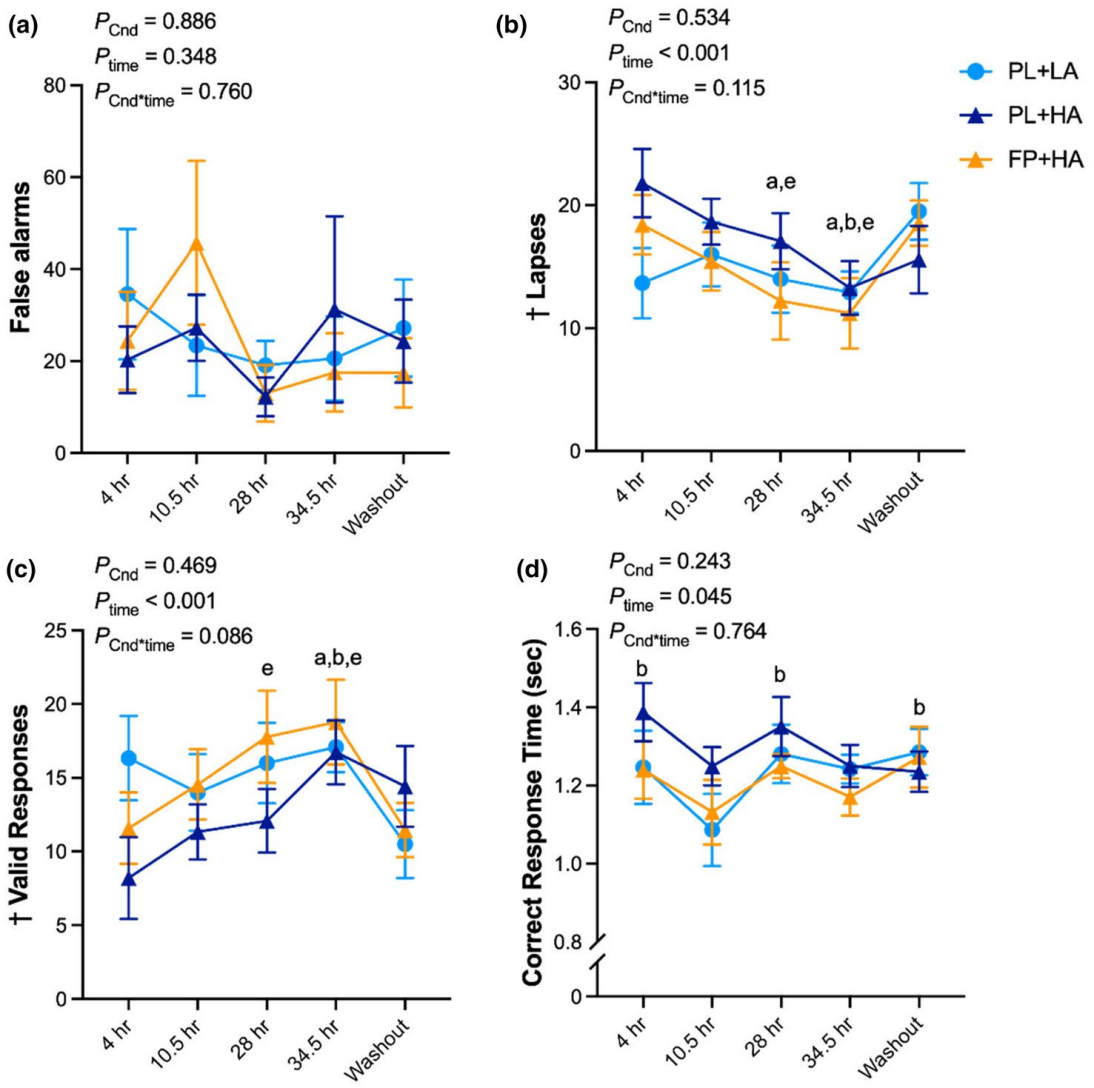
Scanning visual vigilance. The scanning visual vigilance task was administered after 4 h, 10.5 h, 28 h, and 34.5 h of hypobaric chamber residence at low altitude (LA; 500 m) or high altitude (HA; 4300 m) and 36 h post‐chamber (Washout) with daily consumption of a fiber and polyphenol (FP) or matched placebo (PL) snack bar (*n* = 12). Line graph represents raw data as mean and standard error. No significant condition or interaction effects were observed. ^†^Carryover effect identified; data should be interpreted with caution. Letters represent least significant difference post hoc pairwise comparisons for the main effect of time. a = significantly (*p* < 0.05) different than 4 h; b = significantly different than 10.5 h; e = significantly different than Washout. Exact *p* values are presented within the figures for the main effect of experimental condition (*p*_Cnd_), main effect of time (*p*_time_), and their interaction (*p*_Cnd*time_).

There were no significant differences in false alarms, lapses, valid responses, or mean correct response times across experimental conditions (Figure 8a–c; Figure S9a–c). A main effect of experimental condition order was identified for the number of lapses and valid responses, suggesting a possible carryover effect.

## 4 DISCUSSION

This study was designed to test the efficacy of fermentable fiber and polyphenol supplementation targeting the gut microbiota for preventing decrements in mood and cognitive performance following rapid ascent to simulated 4300 m. In prior analyses, we demonstrated that the high altitude environment alone (i.e., without FP supplementation) decreased peripheral oxygen saturation (SpO_2_; LA: ~96% vs. HA: ~71%), caused acute mountain sickness in 54% of the CC cohort, increased intestinal permeability, and increased circulating interleukin‐6, interleukin‐1 receptor antagonist, and cortisol concentrations (Karl et al., 2025). The current findings expand on those observations by demonstrating that the high‐altitude environment also elicited mood disturbances. We previously reported that FP supplementation increased *Bifidobacterium* spp. relative abundance, reduced gut microbiota α‐diversity and colonic pH, and prevented or attenuated hypobaric hypoxia‐induced decrements in intestinal permeability, SpO_2_ and interleukin‐6, but worsened gastrointestinal symptoms and acute mountain sickness (Karl et al., 2025). Herein we report that FP also attenuated altitude‐induced increases in anger, which was observed at high altitude with placebo but not with FP supplementation. However, that benefit was accompanied by elevated confusion. An effect of FP supplementation on cognitive performance was not observed. However, the high altitude environment did not elicit expected decrements in cognitive performance, which limited the ability to detect an effect of FP supplementation. A novel finding was that despite no observed changes in risk‐taking behavior, altitude exposure decreased self‐reported willingness to take risks, which has not been previously reported. Collectively, these findings suggest rapid ascent to simulated 4300 m increases negative mood states and decreases risk propensity. The fermentable fiber and polyphenol formulation tested may attenuate increases in anger but does not appear to alleviate elevations in mood disturbance, tension, and fatigue that occur with rapid ascent to simulated high altitude.

While anger was only elevated during the PL+HA, significant elevations in total mood disturbance (~350%), tension (~60%), fatigue (~260%) and depression (170%) occurred independent of supplementation following rapid ascent to 4300 m versus 500 m. These changes align with known altitude‐induced mood decrements, which typically emerge within 1–4 h, peaking at 18–28 h, and return to baseline within 43–52 h (Shukitt & Banderet, 1988). In a similar hypobaric chamber study, tension increased ~40% within 2 h at 4200 m and ~78% at 4700 m, with additional fatigue (~67%) and confusion (~37%) observed only at 4700 m (Shukitt‐Hale et al., 1998). These findings suggest a dose–response relationship between altitude and mood disturbance, with 4300 m falling within a range where symptoms worsen with increasing altitude (Shukitt‐Hale et al., 1998). Longer exposure at lower altitudes can also affect mood, as soldiers climbing to 3630 m over 7 days exhibited a ~36% decrease in vigor and ~800% increase in fatigue (Shukitt‐Hale et al., 1990). In contrast to previous reports (Shukitt & Banderet, 1988; Shukitt‐Hale et al., 1990, 1998), we did not observe significant effects of altitude on vigor or friendliness. This may be due to previous studies occurring in outdoor settings (Shukitt & Banderet, 1988; Shukitt‐Hale et al., 1990) or during brief (<5 h) chamber exposures (Shukitt‐Hale et al., 1998), whereas the 36‐h confinement in the present study may have masked altitude‐specific changes. Taken together, these data support a time‐ and altitude‐dependent progression of mood disturbances during high altitude exposure (Shukitt‐Hale & Lieberman, 1996).

Altitude also increased anger in the present study, but the FP supplement mitigated that effect. Fermentable fibers (e.g., fructans and galacto‐oligosaccharides) can increase the abundance of *Bifidobacterium* and *Lactobacillus* in the gut microbiota, which in turn can produce neurotransmitters that possess regulatory functions in mood, cognition, and stress regulation (Aslam et al., 2023; Yong et al., 2019). The bacterial fermentation of these fibers also produces metabolites, specifically SCFAs, that can mediate gut–brain communication via immune, endocrine, and vagal pathways and influence mood (Dalile et al., 2019). We previously reported that FP supplementation promoted the growth of beneficial *Bifidobacterium* and *Lactobacillus* species and increased SCFA production in an in vitro large intestine model (Whitman et al., 2024). Further, in the present cohort, FP supplementation increased *Bifidobacterium* relative abundance and decreased colonic pH, suggesting increased SFCA production by the gut microbiota (Karl et al., 2025). Therefore, feelings of anger at simulated 4300 m may have been influenced by gut microbiota‐derived SCFA, neurotransmitters, or other metabolites following consumption of the FP supplement, although further research is needed to replicate this finding and demonstrate causality. Additionally, oxidative stress resulting from elevated levels of reactive oxygen species (ROS) and other free radicals has been observed at high altitudes and in depression (Hano & Tungmunnithum, 2020; Hritcu et al., 2017; Pena et al., 2022). Preclinical studies have demonstrated that flavonoids, a class of bioactive polyphenols, exhibit antidepressive effects through various mechanisms including neurogenesis, neurotransmitters, modulation of receptors, and antioxidant actions (Hritcu et al., 2017; Pannu et al., 2021). Although we did not observe a significant effect of FP supplementation on depression in the present study, research suggests a positive association between depression and anger (Luutonen, 2007) and these mood states were correlated in the present study. Increased anger was also associated with decreased energy as measured by EVAR, a common symptom of depression (Chang et al., 2022). Whether anger is a causal factor of depression or depression contributes to impaired anger regulation remains uncertain (Luutonen, 2007). Therefore, it is plausible that the effects of FP supplementation on anger at simulated 4300 m could occur through similar but distinct mechanisms than those of polyphenols on depression, such as through neutralizing free radicals via antioxidant actions.

A novel finding of the present study was the impact of simulated 4300 m on self‐reported willingness to take risks. Hypoxia has been shown to both increase (Pighin et al., 2012, 2014) and decrease (Heinrich et al., 2019; Niedermeier et al., 2017) risk‐seeking behavior; however, this study was the first to examine attributes of risk propensity. Ratings of total risk propensity, energy, self‐control, and invincibility were lower at simulated 4300 m compared to 500 m independent of supplementation. Despite a tendency for FP supplementation to increase risk‐seeking behavior, assessed by the BART, results from the present study suggest an overall decrease in willingness to take risks under hypoxic conditions. These findings align with previous reports in which participants made fewer risky decisions at altitudes >3800 m during the Game of Dice Task (Niedermeier et al., 2017). In contrast, Heinrich et al. (2019) did not observe significant differences in BART scores between sea level and high altitude (38,000 m). This discrepancy may be attributed to differences in the monetary reward pump ($0.01 vs. $1.00), as the magnitude of the risk‐seeking reward can drive differential responses in risk‐taking behavior (Xu et al., 2018). Decision type may also influence risk‐taking behavior. Pighin et al. (2014) and Pighin et al. (2012) demonstrated that individuals exhibit increased risky behavior under mild hypoxia (3000 m) specifically to avoid loss (e.g., lose $5), but not in situations to secure gain (e.g., keep $5). Therefore, risk‐seeking behavior under hypoxic conditions may differ based on decisions involving gains versus losses and the magnitude of the reward. However, the present findings suggest that attributes such as energy, self‐control, and invincibility may contribute to changes in risk propensity at altitude.

No evidence of impaired cognitive performance following rapid ascent to simulated 4300 m was observed. That result was contrary to our hypothesis and previous reports (Aboouf et al., 2023; Shukitt‐Hale et al., 1998; Shukitt‐Hale & Lieberman, 1996; Virues‐Ortega et al., 2004) wherein cognitive performance commonly worsens as altitude increases, with slower reaction times typically observed at >3500 m, as well as subsequent impairments in spatial and working memory (>4000 m) and memory retrieval (>5000 m) observed at higher altitudes (Aboouf et al., 2023). The absence of decrements in cognitive performance at altitude may be attributed to several factors. First, the sample size in the present study was smaller than that of previous studies (Shukitt & Banderet, 1988; Shukitt‐Hale et al., 1998) and may increase the risk of type II error. Second, both 500 m and 4300 m conditions required confinement in the same small chamber, which may have influenced cognitive outcomes independent of altitude. Third, the unbalanced attrition resulted in a completer cohort wherein the FP+HA condition preceded the PL+LA condition for all but one volunteer. This could result in an underestimation of the effects of hypobaric hypoxia on study outcomes, although that concern is reduced by the general consistency of HA‐associated effects in the ITT and CC analyses. Fourth, previous studies have shown that self‐report mood surveys are more sensitive to stressors than cognitive measures (Caldwell et al., 2004, 2019). Observations of altered mood states, but not cognitive performance, in the present study suggest the mood surveys were more sensitive to hypobaric hypoxia and FP supplementation than cognitive tasks. Importantly, the cognitive tasks used in this study have been previously shown to be sensitive to stress‐related impairments (Lieberman et al., 2005), suggesting the lack of effect was not due to task simplicity. It is possible that participant motivation was low, which could influence task performance; however, motivation was not directly assessed in this study. Together, the small sample size, study environment, attrition, and the sensitivity of the measures may have collectively contributed to the absence of cognitive impairment observed at high altitude and ultimately limited our ability to detect the effects of the FP supplement on cognitive function.

Study strengths include the randomized, double‐blind, placebo‐controlled design, the well‐controlled hypobaric chamber environment, and the controlled diet provided to participants. While these strengths allowed us to isolate the effects of hypobaric hypoxia and dietary supplementation on study outcomes, they do reduce the generalizability to real‐world high‐altitude environments. Several methodological limitations should also be considered when interpreting these results. Although a 14‐day prebiotic supplementation period is sufficient to induce changes in the gut microbiome (Van Harsselaar et al., 2024), it may have been too short to produce measurable effects on cognitive performance or mood, as other studies have observed such effects following 4–12 weeks of supplementation (Serra et al., 2019). Similarly, the 1‐week washout between supplementation periods may not have been sufficient to eliminate potential carryover effects. While the washout duration was based on evidence that substantial diet‐induced shifts in gut microbiota typically resolve within 1 week after an intervention ends (David et al., 2014), more recent best practice recommendations suggest a 4‐week washout to minimize potential carryover effects (Diacova et al., 2025). The absence of a FP+LA condition may also be considered a limitation. However, including a fourth condition was not logistically realistic, and it was considered unlikely that the FP intervention would have measurable cognitive effects in an unstressed healthy population (Dalile et al., 2025). In addition, whether the observed effects on anger at altitude are attributed to the fermentable fiber ingredients, the polyphenol ingredients, or their combination could not be determined. Lastly, this study experienced a high attrition rate largely due to the COVID‐19 pandemic; that attrition could have resulted in an underestimation of the effects of hypobaric hypoxia and dietary supplementation on study outcomes. However, both the CC and ITT analyses are reported to help address any biases that may have resulted from that attrition.

In summary, the present study suggests that consuming a supplement containing fermentable fibers and polyphenols may attenuate increases in anger, but have little impact on elevated tension and fatigue, or other aspects of cognitive performance at high altitude. Future studies should investigate the individual effects of fermentable fibers and polyphenols on mood and risk propensity at high altitude to discern the individual contributions and examine other stressors that degrade mood and cognitive function through mechanisms that differ from those at simulated 4300 m for a more direct evaluation of FP supplementation efficacy.

## AUTHOR CONTRIBUTIONS

J.P.K. conceived and designed the research; H.S.F., P.J.N., G.E.G., and J.P.K. performed experiments; M.E.B., G.E.G., and J.P.K. analyzed data; M.E.B., G.E.G., H.R.L., and J.P.K. interpreted results of experiments; M.E.B. prepared figures and drafted the manuscript; M.E.B., H.S.F., P.J.N., G.E.G., H.R.L., and J.P.K. edited and revised the manuscript; M.E.B., H.S.F., P.J.N., G.E.G., H.R.L., and J.P.K. approved the final version of the manuscript.

## CONFLICT OF INTEREST STATEMENT

No conflicts of interest, financial or otherwise, are declared by the authors.

## ETHICS STATEMENT

The study was centrally approved by the Headquarters U.S. Army Medical Research and Development Command Institutional Review Board and conducted in adherence with the provisions of 32 CFR Part 219.

## DISCLAIMER

The opinions or assertions contained herein are the private views of the authors and are not to be construed as official or reflecting the views of the Army or the Department of Defense. Any citations of commercial organizations and trade names in this report do not constitute an official Department of the Army endorsement or approval of the products or services of these organizations. Funded by the Defense Health Program, US Army Medical Research and Development Command, Military Operational Medicine Research Program, and appointment to the US Army Research Institute of Environmental Medicine administered by the Oak Ridge Institute for Science and Education through an interagency agreement between the US Department of Energy and the US Army Medical Research and Development Command.

## Supporting information


**Figures S1–S9**.

**Tables S1–S8**.

## Data Availability

Data are available upon reasonable request and pending ethical and legal approvals.
