# Supplementary material for: Effects of fermentable fiber and polyphenol supplementation on mood and cognition in adults during hypobaric hypoxia exposure
Source: Physiol Rep. 2025 Sep 10;13(17):e70541. doi: 10.14814/phy2.70541 (PMC12422811; doi:10.14814/phy2.70541)
Supplement: Supplementary file 1 — Figures S1–S9. [file PHY2-13-e70541-s001.docx]

# Randomized n = 33

Phase 1: PL+LA

Phase 2: FP+HA

Phase 3: PL+HA

# n = 8

Phase 1: PL+HA

Phase 2: PL+LA

Phase 3: FP+HA

# n = 9

Phase 1: FP+HA

Phase 2: PL+HA

Phase 3: PL+LA

# n = 16

Withdrawals:

COVID (n = 3)1

Personal reasons (n = 2) Study-related AE (n = 2)2

Completed Phase 1

# (ITT cohort; n = 8)

Unrelated illness (n = 1)3 Study-related AE (n = 2)4,5

Completed Phase 1

# (ITT cohort; n = 9)

Withdrawals:

COVID (n = 6)1

Personal reasons (n = 1) Relocation (n = 1)

Completed Phase 1

# (ITT cohort; n = 9)

Withdrawals:

COVID (n = 3)1

Study-related AE (n = 1)5

Completed Phase 2

# n = 7

Completed Phase 2

# n = 1

Completed Phase 2

# n = 5

Study-related AE (n = 1)3.4

Completed Phase 3

# n = 8

Completed Phase 3

# n = 1

Completed Phase 3

# n = 5

Completer cohort

# n = 7

Completer cohort

# n = 1

Completer cohort

n = 5

**Figure S1.** **CONSORT diagram.** This randomized, placebo-controlled crossover clinical trial consisted of three phases. Each phase included a 14-day supplementation period followed by a ≥1-week washout period. Participants consumed one of two types of supplemental snack bars during each supplementation period: fermentable fiber and polyphenol (FP, consumed during one phase) or a matched placebo (PL, consumed during two phases). During days 13-14 of each phase, participants resided in a hypobaric chamber for 36 hours simulating either low altitude (LA; 500 m [720 mm Hg], one phase) or high altitude (HA; 4300 m [460 mm Hg], two phases). The combination of supplementation and altitude resulted in three experimental conditions: PL+LA, PL+HA, and FP+HA. This figure was originally published by Karl et al., 2025 in *American Journal of Physiology-Regulatory, Integrative and Comparative Physiology*, and is reproduced here with permission for reference.

^1^Unavailable after institute shutdown due to coronavirus (COVID)-19 pandemic.

^2^Gastrointestinal symptoms during consumption of study diet and intervention.

^3^Participant missed Phase 2 chamber residence due to illness but completed all of Phase 3.

^4^Participants completed one full day of chamber residence during the phase but remained on study diet through intervention period and were included in completer cohort.

^5^Symptoms consistent with acute mountain sickness.

AE, adverse event; ITT, intention-to-treat.

**
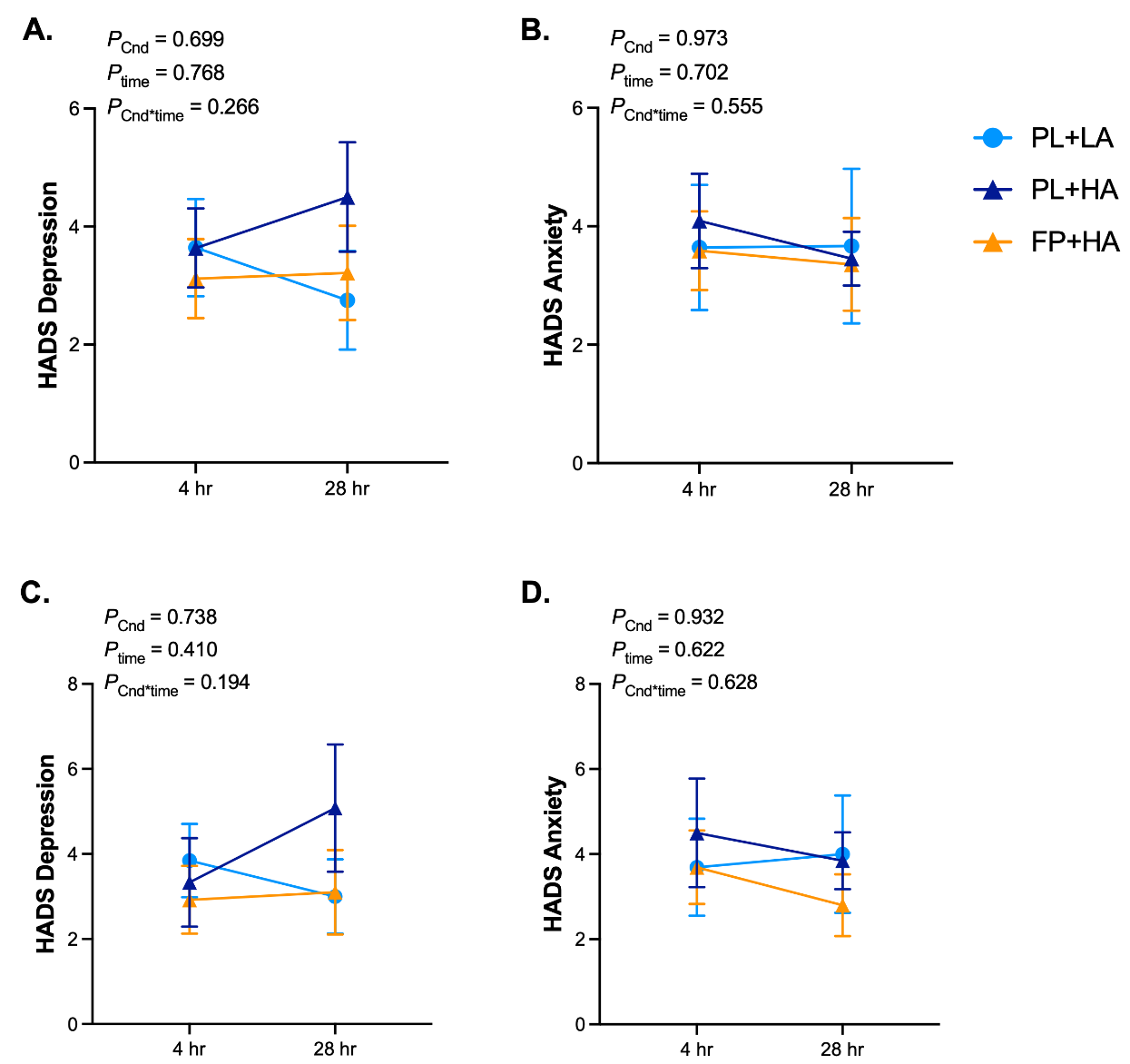
**

**Figure S2. Hospital Anxiety and Depression Scale (HADS) in Intention-to-treat (ITT, n=26) cohort (A-B) and Complete Case (CC, n=13) cohort (C-D).** HADS was completed after 4-hrand 28-hr of hypobaric chamber residence at low altitude (LA) or high altitude (HA) with daily consumption of a fiber and polyphenol (FP) or matched placebo (PL) snack bar. Line graph represents raw data as mean and standard error. Exact p-values are presented within the figures for main effect of experimental condition (*P*_Cnd_), main effect of time (*P*_time_), and their interaction (*P*_Cnd*time_).

**
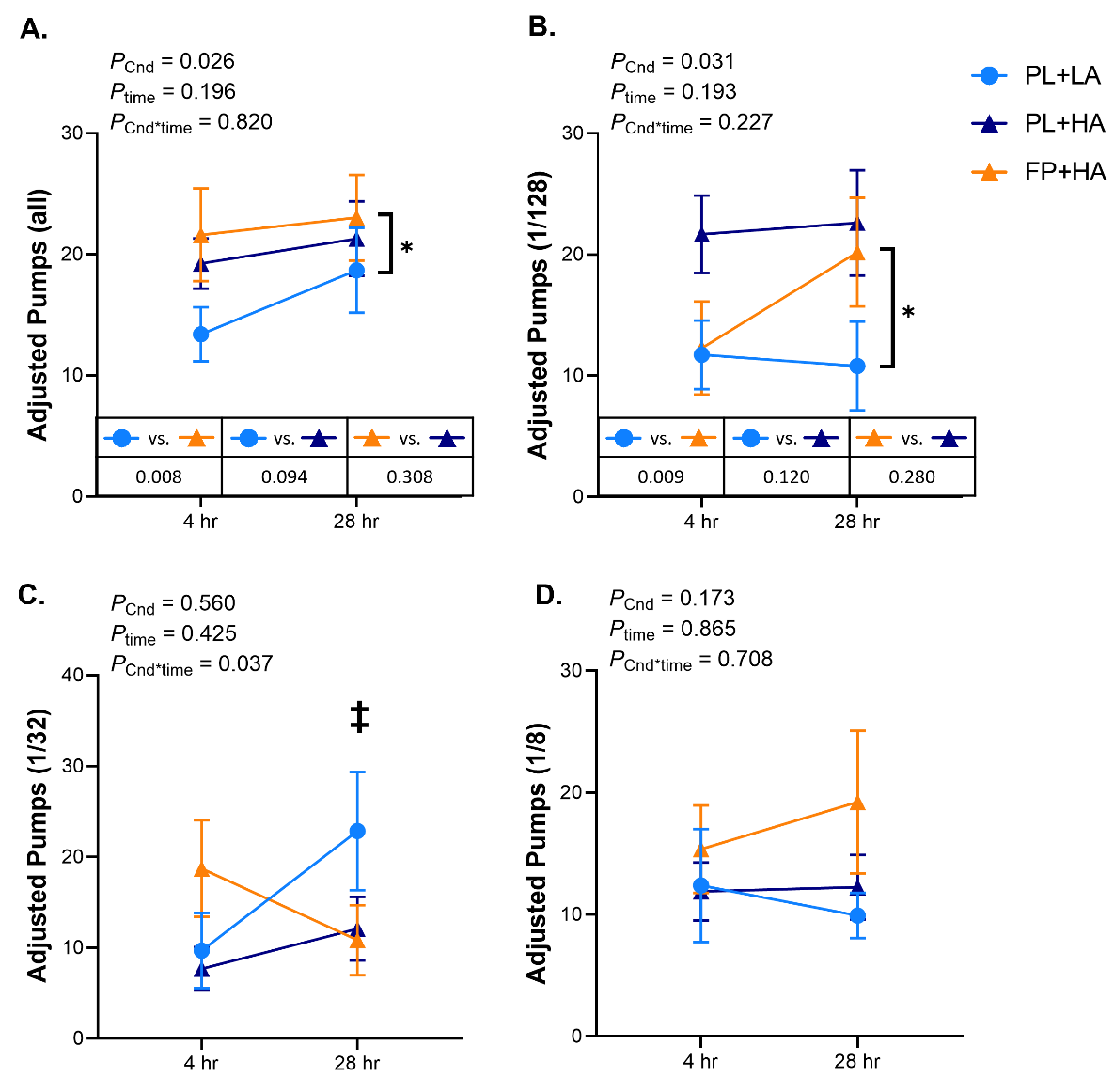
**

**Figure S3. Balloon Analog Risk Task (BART) in Intention-to-treat (ITT, n=25) cohort.** BART was completed after 4-hr and 28-hr of hypobaric chamber residence at low altitude (LA) or high altitude (HA) with daily consumption of a fiber and polyphenol (FP) or matched placebo (PL) snack bar. Line graph represents raw data as mean and standard error. *Main effect of experimental condition (*p* < 0.05) with post-hoc *p*-values presented within the embedded table. ‡ Simple main effect of experimental condition where PL+HA was significantly (*p* = 0.05) different than FP+HA, but not PL+HA, after 28-hr of hypobaric chamber residence. Exact p-values are presented within the figures for main effect of experimental condition (*P*_Cnd_), main effect of time (*P*_time_), and their interaction (*P*_Cnd*time_).

**
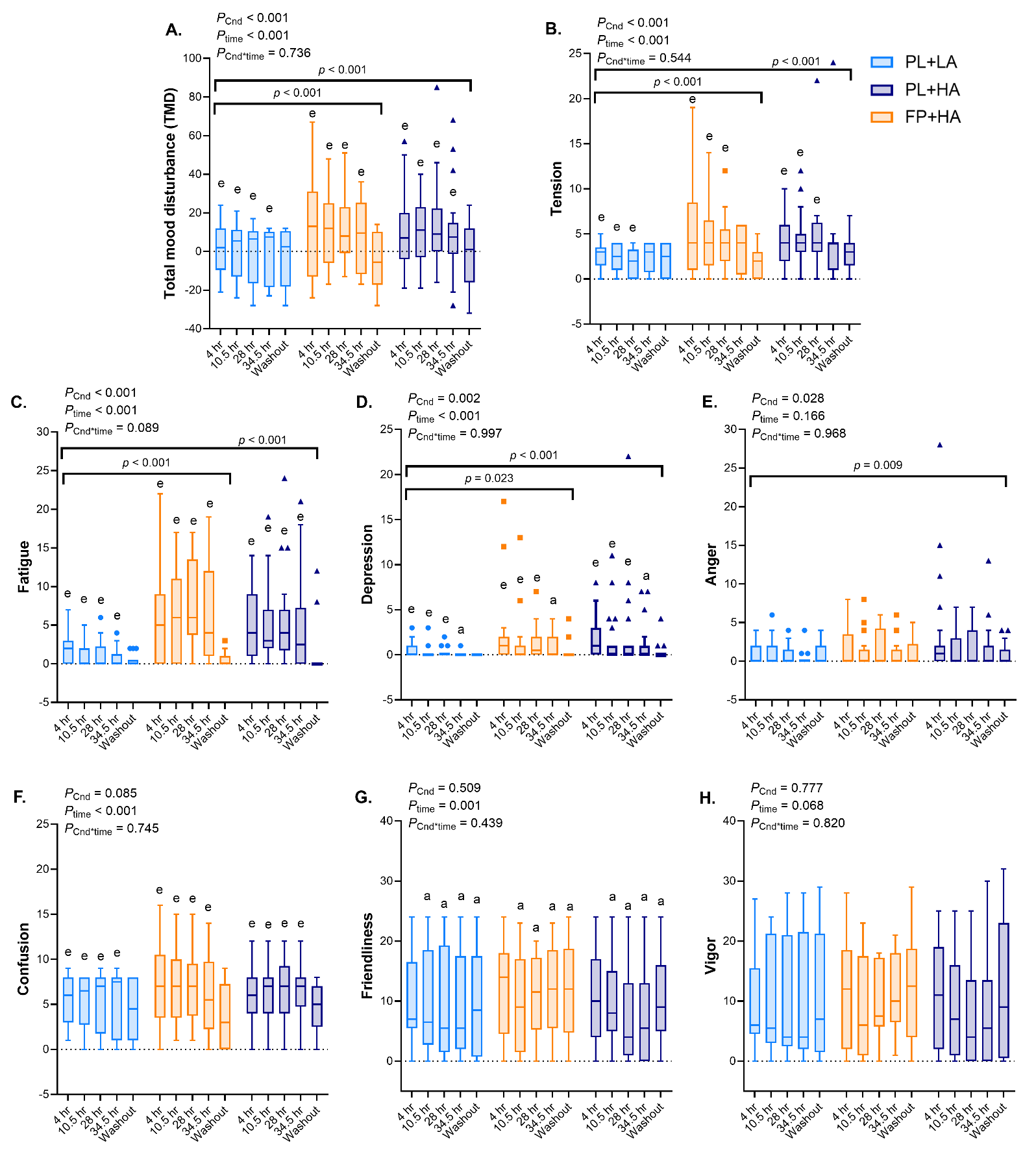
**

**Figure S4. Profile of Mood States (POMS) in Intention-to-treat (ITT) cohort.** Profile of mood states was measured after 4-hr, 10.5-hr, 28-hr, and 34.5-hr of hypobaric chamber residence at low altitude (LA) or high altitude (HA) and 36 hours post-chamber (Washout) with daily consumption of a fiber and polyphenol (FP) or matched placebo (PL) snack bar (n=26). All POMS subscales except friendliness were square root transformed to meet assumptions. Boxes indicate raw data median and interquartile range. Whiskers extend to 1.5 times the interquartile range, or to the minimum and maximum if no outliers are present. Outliers are presented as individual data points. No significant interaction effects were observed. Post hoc *p*-values following significant main effect of experimental condition (*) are presented above horizontal bars. Letters represent least significant difference *post-hoc* pairwise comparisons for main effect of time. a= significantly (*p* < 0.05) different than 4-hr; e = significantly different than Washout (main effect of time). Exact p-values are presented within the figures for main effect of experimental condition (*P*_Cnd_), main effect of time (*P*_time_), and their interaction (*P*_Cnd*time_).

**
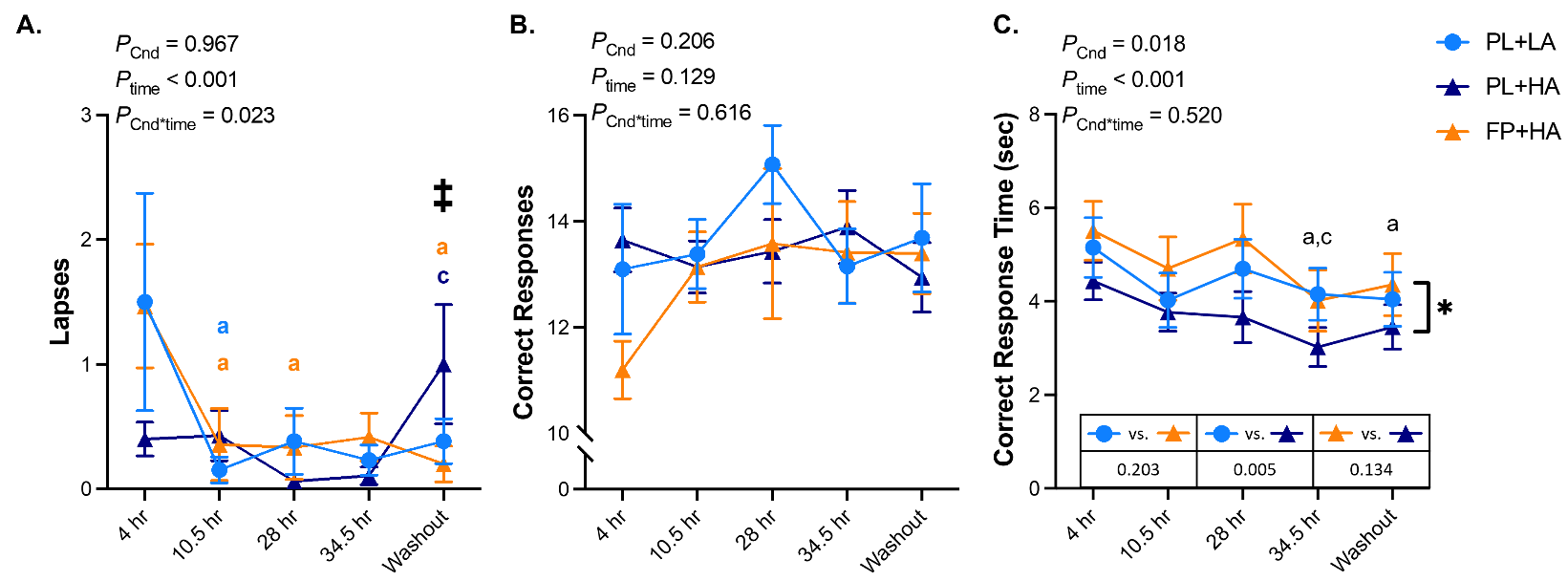
**

**Figure S5. Matching to Sample in Intention-to-treat (ITT) cohort.** The Matching to Sample test was administered after 4-hr, 10.5-hr, 28-hr, and 34.5-hr of hypobaric chamber residence at low altitude (LA) or high altitude (HA) and 36 hours post-chamber (Washout) with daily consumption of a fiber and polyphenol (FP) or matched placebo (PL) snack bar (n=24). Line graph represents raw data as mean and standard error. ‡ Simple main effect of experimental condition where PL+HA was significantly different than PL+LA (*p* = 0.039) and FP+HA (*p* = 0.011). *Main effect of experimental condition (*p* < 0.05) with post-hoc *p*-values presented within the embedded table. Color letters represent least significant difference *post-hoc* comparisons for simple main effect of time. Black letters represent least significant difference *post-hoc* pairwise comparisons for main effect of time. a= significantly (*p* < 0.05) different than 4-hr; b= significantly different than 10.5-hr; c= significantly different than 28-hr; e= significantly different than Washout. Exact p-values are presented within the figures for main effect of experimental condition (*P*_Cnd_), main effect of time (*P*_time_), and their interaction (*P*_Cnd*time_).

**
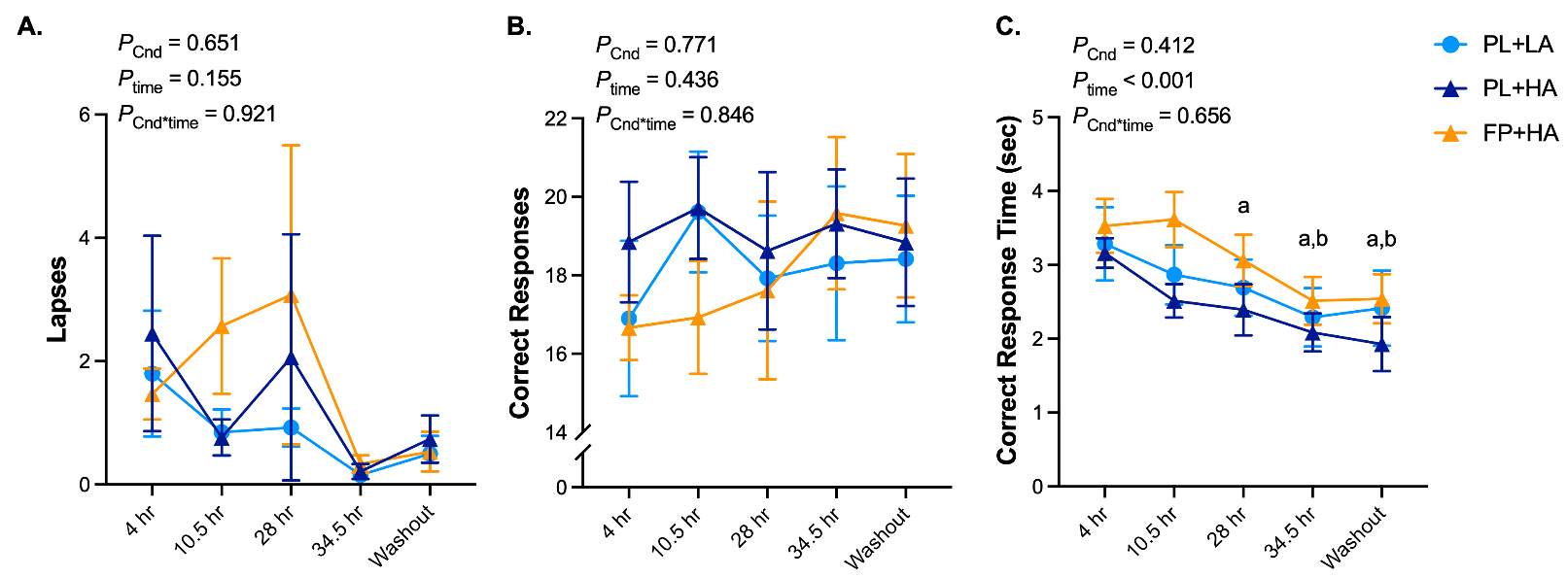
**

**Figure S6. Grammatical Reasoning in Intention-to-treat (ITT) cohort.** The Grammatical Reasoning test was administered after 4-hr, 10.5-hr, 28-hr, and 34.5-hr of hypobaric chamber residence at low altitude (LA) or high altitude (HA) and 36 hours post-chamber (Washout) with daily consumption of a fiber and polyphenol (FP) or matched placebo (PL) snack bar (n=24). Line graph represents raw data as mean and standard error. No significant condition or interactions effects were observed. Letters represent least significant difference *post-hoc* pairwise comparisons for main effect of time. a= significantly (*p* < 0.05) different than 4-hr; b = significantly different than 10.5-hr. Exact p-values are presented within the figures for main effect of experimental condition (*P*_Cnd_), main effect of time (*P*_time_), and their interaction (*P*_Cnd*time_).

**
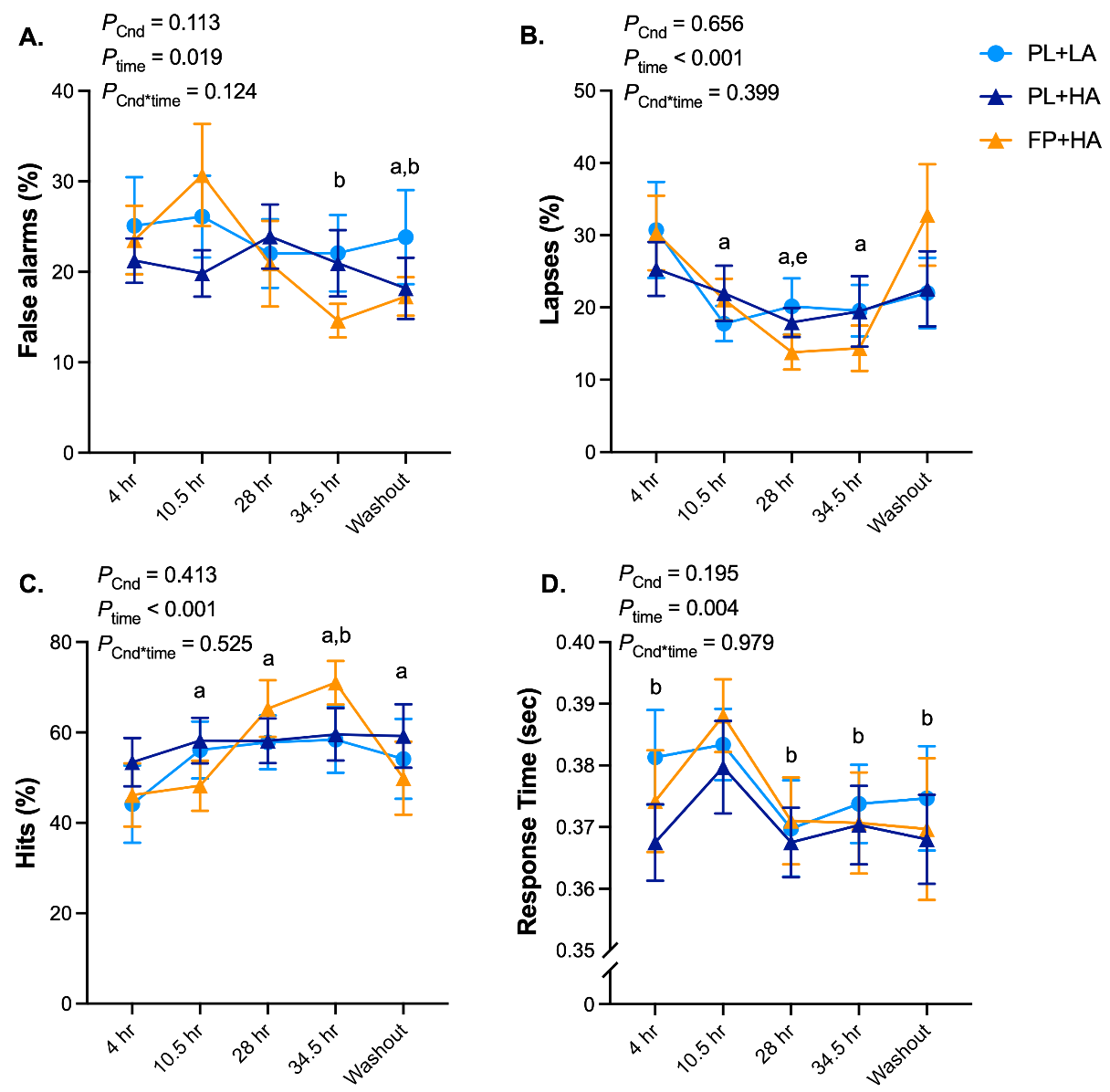
**

**Figure S7. Psychomotor Vigilance Test (PVT) in Intention-to-treat (ITT) cohort.** Psychomotor vigilance was assessed after 4-hr, 10.5-hr, 28-hr, and 34.5-hr of hypobaric chamber residence at low altitude (LA) or high altitude (HA) and 36 hours post-chamber (Washout) with daily consumption of a fiber and polyphenol (FP) or matched placebo (PL) snack bar (n=24). Line graph represents raw data as mean and standard error. No significant condition or interactions effects were observed. Letters represent least significant difference *post-hoc* pairwise comparisons for main effect of time. a= significantly (*p* < 0.05) different than4-hr; b= significantly different than 10.5-hr; e= significantly different than Washout. Exact p-values are presented within the figures for main effect of experimental condition (*P*_Cnd_), main effect of time (*P*_time_), and their interaction (*P*_Cnd*time_).

**
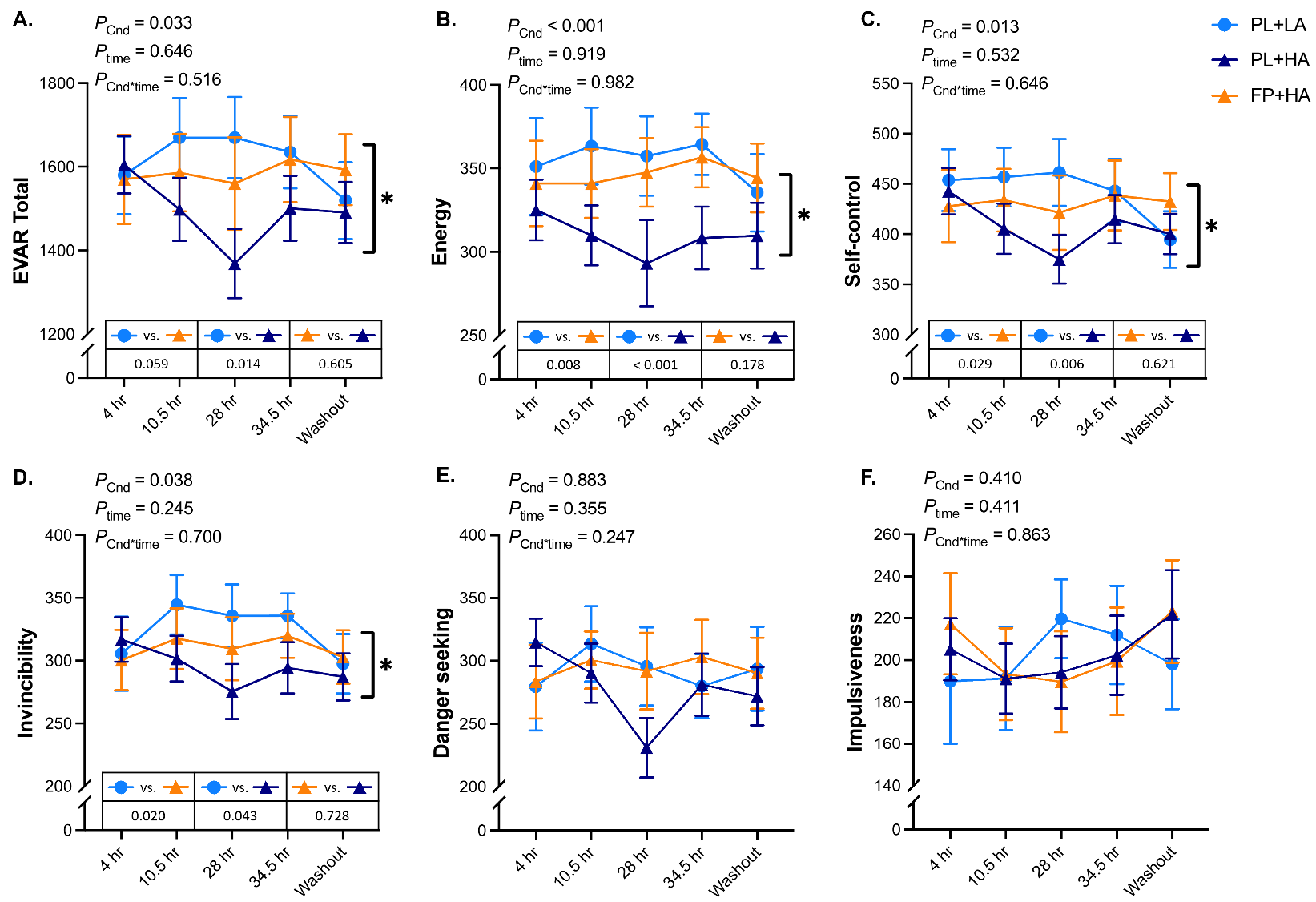
**

**Figure S8. Evaluation of Risks Scale (EVAR) in Intention-to-treat (ITT) cohort.** The EVAR scale was administered after 4-hr, 10.5-hr, 28-hr, and 34.5-hrof hypobaric chamber residence at low altitude (LA) or high altitude (HA) and 36 hours post-chamber (Washout) with daily consumption of a fiber and polyphenol (FP) or matched placebo (PL) snack bar (n=25). Line graph represents raw data as mean and standard error. No significant time or interactions effects were observed. *Main effect of experimental condition (*p* < 0.05) with post-hoc *p*-values presented within the embedded table. Exact p-values are presented within the figures for main effect of experimental condition (*P*_Cnd_), main effect of time (*P*_time_), and their interaction (*P*_Cnd*time_).

**
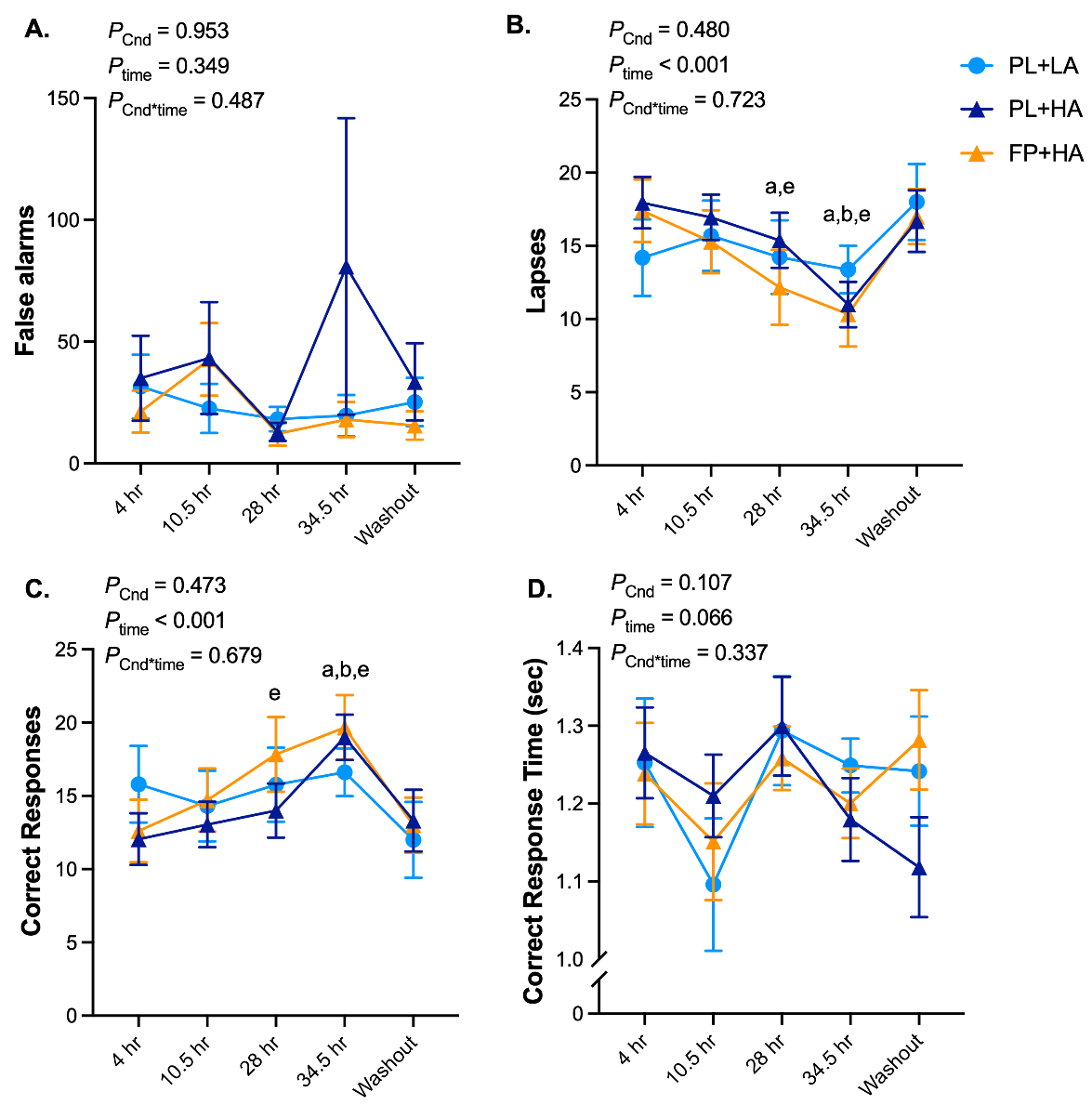
**

**Figure S9. Scanning Visual Vigilance in Intention-to-treat (ITT) cohort.** The Scanning Visual Vigilance task was administered after 4-hr, 10.5-hr, 28-hr, and 34.5-hr of hypobaric chamber residence at low altitude (LA) or high altitude (HA) and 36 hours post-chamber (Washout) with daily consumption of a fiber and polyphenol (FP) or matched placebo (PL) snack bar (n=24). Line graph represents raw data as mean and standard error. No significant condition or interactions effects were observed. Letters represent least significant difference *post-hoc* pairwise comparisons for main effect of time. a= significantly (*p* < 0.05) different than 4-hr; b = significantly different than 10.5-hr; e= significantly different than Washout. Exact p-values are presented within the figures for main effect of experimental condition (*P*_Cnd_), main effect of time (*P*_time_), and their interaction (*P*_Cnd*time_).
